# *Octopus maya* white body show sex-specific transcriptomic profiles during the reproductive phase, with high differentiation in signaling pathways

**DOI:** 10.1371/journal.pone.0216982

**Published:** 2019-05-16

**Authors:** Oscar E. Juárez, Laura López-Galindo, Leonel Pérez-Carrasco, Asunción Lago-Lestón, Carlos Rosas, Anna Di Cosmo, Clara E. Galindo-Sánchez

**Affiliations:** 1 Departamento de Biotecnología Marina, Centro de Investigación Científica y de Educación Superior de Ensenada, Zona Playitas, Ensenada, Baja California, México; 2 Departamento de Innovación Biomédica, Centro de Investigación Científica y de Educación Superior de Ensenada, Zona Playitas, Ensenada, Baja California, México; 3 Unidad Académica Sisal, Universidad Nacional Autónoma de México, Puerto de Abrigo s/n, Sisal, Yucatán, México; 4 Dipartimento di Biologia, Università degli Studi di Napoli Federico II, Napoli, Italia; Shanghai Ocean University, CHINA

## Abstract

White bodies (WB), multilobulated soft tissue that wraps the optic tracts and optic lobes, have been considered the hematopoietic organ of the cephalopods. Its glandular appearance and its lobular morphology suggest that different parts of the WB may perform different functions, but a detailed functional analysis of the octopus WB is lacking. The aim of this study is to describe the transcriptomic profile of WB to better understand its functions, with emphasis on the difference between sexes during reproductive events. Then, validation via qPCR was performed using different tissues to find out tissue-specific transcripts. High differentiation in signaling pathways was observed in the comparison of female and male transcriptomic profiles. For instance, the expression of genes involved in the androgen receptor-signaling pathway were detected only in males, whereas estrogen receptor showed higher expression in females. Highly expressed genes in males enriched oxidation-reduction and apoptotic processes, which are related to the immune response. On the other hand, expression of genes involved in replicative senescence and the response to cortisol were only detected in females. Moreover, the transcripts with higher expression in females enriched a wide variety of signaling pathways mediated by molecules like neuropeptides, integrins, MAPKs and receptors like TNF and Toll-like. In addition, these putative neuropeptide transcripts, showed higher expression in females’ WB and were not detected in other analyzed tissues. These results suggest that the differentiation in signaling pathways in white bodies of *O*. *maya* influences the physiological dimorphism between females and males during the reproductive phase.

## Introduction

The white body (WB) was first described by Cuvier [1] as a “corps glanduleux” surrounding the optic lobes and optic traits with the only function to protect these structures during muscle contraction. Later the WB was considered as site for the formation of leucocytes [2,3]. Cazal and Bogoraze [4] proposed a second function for the WB: “fonction nèphrocytaire”, and both functions were supported by the work of Bolognari [5]. Then Harrison and Martin [6] demonstrated an important role of WB in urine formation; however Young [7], retained that its function was still unclear.

In cephalopods, it is well known that haemocytes are originated within the WB, in fact, several hematopoiesis genes were found to be expressed in the WB of squid *Euprymna tasmanica* [8]. In addition, transcripts associated with immune-related signal transduction pathways were found, as well as other genes of the immune response previously identified in *E*. *scolopes* [9]. Nevertheless, its general similitude with the mammal lymphoid tissue, its lobular morphology, and its glandular appearance suggest that different WB regions may perform different functions [10,11]; but in octopuses, a detailed functional analysis of the WB is lacking. This organ links the nervous and circulatory systems and may play a role in the cooperation between neuroendocrine and immune responses to environmental stimuli [12]. However, the understanding of how these two systems cooperate, remains unclear [12]. Such interaction may be mediated via secretion/reception of hormones, neuropeptides or signaling peptides. The neuroendocrine crosstalk among different lobes of the nervous system, which regulates physiology and reproduction of *O*. *vulgaris*, is well documented [12–16]. However, if the WB takes a place in this crosstalk is poorly understood. Herein, to better understand the WB functions, this study was directed to describe its gene expression, with emphasis in sex-related and reproductive-stage related differences via RNA-Seq. This technology has been successfully implemented for gene discovery and for estimation of gene expression levels in different cephalopod tissues [8,17–21]. In addition, different tissues were compared in terms of gene expression to identify sex-specific and tissue-specific transcripts.

## Method

### Ethics statement

In this study, octopuses were anesthetized with ethanol 3% in sea water at experimental temperatures [22,23] to enable humane killing [24] in consideration of ethical protocols [25], and the animals’ welfare during manipulations [26,27]. Our protocols were approved by the experimental Animal Ethics Committee of the Faculty of Chemistry at Universidad Nacional Autónoma de México (Permit number: Oficio/FQ/CICUAL/099/15). We encouraged the effort to minimize animals stress and the killing of the minimum necessary number of animals for this study.

### Acclimation and experimental design

Female and male octopuses with a body mass that ranged between 400–600 g were captured off the coast of Sisal Yucatán, México, by artisanal fishing fleet and then transported to the Experimental Cephalopod Production Unit at the Multidisciplinary Unit for Teaching and Research (UMDI-UNAM), Sisal, Yucatan, Mexico. Octopuses were acclimated for 10 d with 1:1 sex ratio, in 6 m diameter outdoor ponds provided with aerated natural seawater (26 ± 1°C). The ponds were covered with black mesh reducing direct sunlight to 70% and connected to seawater recirculation systems coupled to protein skimmers and 50 μm bag filters. PVC 50 mm diameter open tubes were offered as refuges in proportion 2:1 per animal. Octopuses were fed individually twice a day with a paste made with squid and crab meat at ratio of 8% of its body weight [28]. Food not ingested, and feces were removed daily. During acclimation octopuses paired freely; then, a group of ten males were sampled before copulation and another group of ten were sampled after copulation. On the other hand, twenty fertilized females were individually reared in 80L tanks in a recirculating aquaculture water system. Each tank was provided with a fiberglass box that serves as refuge for the female and for spawn settling. System temperature was maintained at 24°C, until all females spawned. These conditions were selected because 24°C is the preferred temperature of this species and has been recommended as the best condition for spawning [29,30]. Water was heated by using a 1200W titanium immersion heater connected to a digital temperature sensor, both placed at system reservoir; and was cooled by using air conditioning according to the temperature required.

### White body sampling

Octopus sensibility was minimized before manipulation, by means of an anesthetic procedure that consisted in maintaining animals in 3% alcohol-sea water solution for up to 4 minutes [23]. White body samples were obtained from ten males before copulation (MPRE) and from ten males after copulation (MPOS). In the case of females, ten WB samples were obtained before spawning (FPRE) and another ten samples after spawning (FPOS). All WB samples were taken from the region adjacent to the optic lobe. Additional samples from different tissues (including hearts and gonads) were obtained from the same experimental individuals, for further analyses. All tissue samples were preserved in RNAlater solution until RNA extraction.

### RNA sequencing

Total RNA was extracted from 30 mg of WB tissue using the RNeasy extraction kit (Qiagen) following manufacturer instructions. RNA was quantified with NanoDrop 2000 spectrophotometer (Thermo Scientific) and quality was assessed using the Bioanalyzer Instrument 2100 (Agilent Technologies). Samples that presented an RNA integrity number (RIN) equal or higher than 7 were used for sequencing. Using equal amounts of RNA from the three individuals with higher RIN in each experimental condition, paired-end cDNA libraries were prepared using the TruSeq DNA Sample Preparation Kit v2 (Illumina), following the manufacturers protocol. Subsequently, cluster generation and DNA sequencing were performed in MiSeq sequencing system (Illumina) to obtain reads of 250 bp long.

### De novo transcriptome assembly

The quality of raw sequence data was assessed with FastQC v. 0.11.6 (Babraham Bioinformatics). Low quality reads were discarded with Trimmomatic v0.35 software [31] keeping those with Phred score above 28, for subsequent analysis. Reads from all libraries (reads accession number: SRR8049182, SRR8049183, SRR8049184, SRR8049185) were de novo assembled using Trinity v2.4.0 [32] using default parameters (assembly accession number: GHBT00000000; BioProject: PRJNA496073).

### Differential expression analysis

The transcriptome assembled with all female and male reads was used as reference to estimate the abundance of the transcripts for each library: FPRE, FPOS, MPRE and MPOS. The reads from each library were aligned back to the reference transcriptome by using Bowtie2 v2.3.2 [33], followed by quantification and normalization (fragments per Kilobase million, FPKM) with RSEM v1.3.0 [34]. A matrix including the FPKM of all the libraries was analyzed to identify the differentially expressed (DE) transcripts (false discovery rate, FDR < 0.01, fold change > 2) with edgeR package (Bioconductor) in R software [35] using a dispersion value of 0.1, which is suitable for non-replicate data [36]. The DE transcripts were arranged in clusters according to their expression pattern and were represented with a heatmap. These analyses were performed using the Perl and R scripts included in Trinity v2.4.0.

### Functional annotation of transcripts

The reference transcriptome (assembled from the reads of both sexes) was analyzed with BLASTx [37] to find homologs within UniProt Release 2017_12 database with an e-value < E-05 filter. The Gene Ontology (GO) annotations were obtained based on the UniProt IDs using Blast2GO v4.1 with an e-value filter of 1E-08 [38]. The GO annotations for each transcript were analyzed to identify the best-represented biological processes detected in the reference transcriptome, based on the number of sequences included in each GO category. On the other hand, a Fisher exact test (FDR < 0.05) was implemented to identify the enriched biological processes in each library, using the annotated DE transcripts as test-set and the reference transcriptome as background in Blast2GO v4.1. In the same way, an additional enrichment of metabolic pathways from Kyoto Encyclopedia of Genes and Genomes (KEGG) database [39] was performed, using DAVID 6.8 [40] with the annotated DE transcripts as test-set and the assembled reference transcriptome as background. From the enriched categories, transcripts were selected for further analysis to confirm the annotation results. These transcripts were analyzed with TransDecoder v5.5.0 [41] to identify their longest open reading frames (ORF), and to predict their coding sequences and encoded peptides. Then, BLASTp searches against the non-redundant (nr) protein database were performed online (https://blast.ncbi.nlm.nih.gov/Blast.cgi) for the predicted encoded peptides.

### Phylogenetic analysis of relevant DE transcripts

A phylogenetic analysis was performed to corroborate the BLASTx results of DE transcripts involved in relevant biological processes. A nucleotide sequence alignment was built, including all the mRNA sequences from mollusks available in the Genbank nucleotide database, corresponding to the putative gene analyzed. Nucleotide and codon alignments were carried out with the ClustalW 2.0 algorithm [42]; then the alignments were analyzed to find the nucleotide substitution model that best described the data, using the Maximum Likelihood method (ML). The model with lowest Bayesian Information Criterion score was considered the best [43]. The phylogenetic relationship among the sequences was inferred by using the ML method based on the General Time Reversible model [43]. Initial tree for the heuristic search was obtained automatically by applying Neighbor-Join and BioNJ algorithms to a matrix of pairwise distances estimated using the Maximum Composite Likelihood approach, and then selecting the topology with superior log likelihood value. The bootstrap consensus tree inferred from 200 replicates was taken to represent the evolutionary history of the sequences analyzed [44]. Evolutionary analyses were conducted in MEGA7 [45].

### Validation of DE transcripts via qPCR

A quantitative real-time PCR analysis was performed to validate the sex-related and tissue specific expression of transcripts, using additional tissues (systemic heart and testis). RNA samples were treated with RQ1 RNase-free DNase (Promega) before cDNA synthesis. The cDNA synthesis was carried out with the Improm II Reverse Transcription System (Promega) following manufacturer's instructions starting with 1μg of RNA from each sample. Primers for qPCR were designed with Primer3 [46] based on the selected transcript sequences (Table 1). To calculate the primer amplification efficiency, a standard curve was built including five stepwise dilutions of the cDNA samples with a constant dilution factor of 1:5, using nuclease-free water. The amplification reaction included 7μL of B-R SYBR Green Super Mix (Quanta Biosciences) and 3 μL of each cDNA dilution. Cq values were obtained in a C1000 Touch Thermal Cycler including the CFX96 Real Time System (Bio-Rad) with an amplification program consisting in: 2 min at 95°C, 35 cycles of 30s at 95°C, 30s at 57°C, 30s at 72°C and plate read, followed by 5 min at 72°C, 10s at 95°C, 30s at 65°C, 60 cycles of 5s at 65°C + 0.5°C/cycle and plate read. The amplification efficiency of the primers (E) was calculated as E = [10(-1/k)-1], with k representing the slope of the standard curve [47]. For relative expression analysis, all reference and target transcripts were amplified by qPCR reactions within 96-well plastic plates (including positive and non-template controls) using the same instrument, reagents and amplification program as performed for amplification efficiency calculation. Nine biological replicates per sex and tissue were analyzed individually and by triplicate. The relative expression of target transcripts in each group, were estimated using the ΔΔ Cq method in Qbase+ 3.0 software [48] using as reference the heterogeneous nuclear ribonucleoprotein D like (HNRNPD) and the V-type proton ATPase subunit D (VATD) putative genes. Reference genes were validated previously using geNorm [49] and NormFinder [50]. For statistical analysis of relative gene expression values from RT-qPCR, one-way ANOVA and Tukey’s multiple comparisons test were performed (P < 0.05). Moreover, Kruskal Wallis and Dunn’s post hoc tests (P < 0.05) were used when assumptions of normality (Shapiro-Wilk) were not satisfied. To test if the relative expression values obtained by RNA-Seq and qPCR were consistent, a Spearman correlation was performed (P < 0.05). Statistical analyses were performed using Statistica7 software [51].

**Table 1:** Target and reference genes, primers sequences and melting temperatures for qPCR analysis.

Putative encoded protein	Tm°C	Forward	Reverse
**Targets**			
C-Jun-amino-terminal kinase-interacting protein 4 (Spag9)	57–60	GAGCTTCAGATGGCCAATGG	TTGCAGCCACACCATACATG
Corticotropin-releasing factor receptor 2	57–60	CACCAAAGCACCCTTGACAG	CCATTGTGTGCCTGTATTTCTG
Estradiol 17-beta-dehydrogenase 8	57–60	CAGTTGGGAAAGCATGGTGG	CAGTGTCTGCCAGGTGTTTG
Estrogen receptor	57–60	AGGTAGCCAAAGGAAGGAGAG	AAACGCTGACTCTTTGCTGG
Putative neuropeptide (FMRF-amide like)	57–60	TTATTTCCATCAAAGCTG	AGATCTGATCATGGCAGT
Piwi-like protein 1	57–60	GCGGTCTGAAAGGTTGTACG	GACCATTACCACGACTCTGC
ATP-dependent RNA helicase DDX4 (Vasa homolog)	57–60	GCCATTAGATCACGACCAGC	GAAGCCCAATTCCAGCAAAAG
Zonadhesin	57–60	CCACATTATTCGGGCTTCAGG	GTCAACACCGGCCTTTGTAC
**Reference**			
V-type proton ATPase subunit D	57	TGAAATACGGTGCAAGAGGGG	ACTGATTCCCAAGAGCCATCC
Heterogeneous nuclear ribonucleoprotein D-like	60	GTTCTCGTGGATTTGCTCGC	TCCAGAGGTTTTGGTTTTGTCC

## Results

### Transcriptome assembly

For the reference transcriptome 75,265 unigenes and 90,435 isoforms were reconstructed, showing an average contig length of 622 bases, an N50 of 875 bases and a total of 56,280,138 bases assembled.

### Differential expression analysis

**Fig 1:**
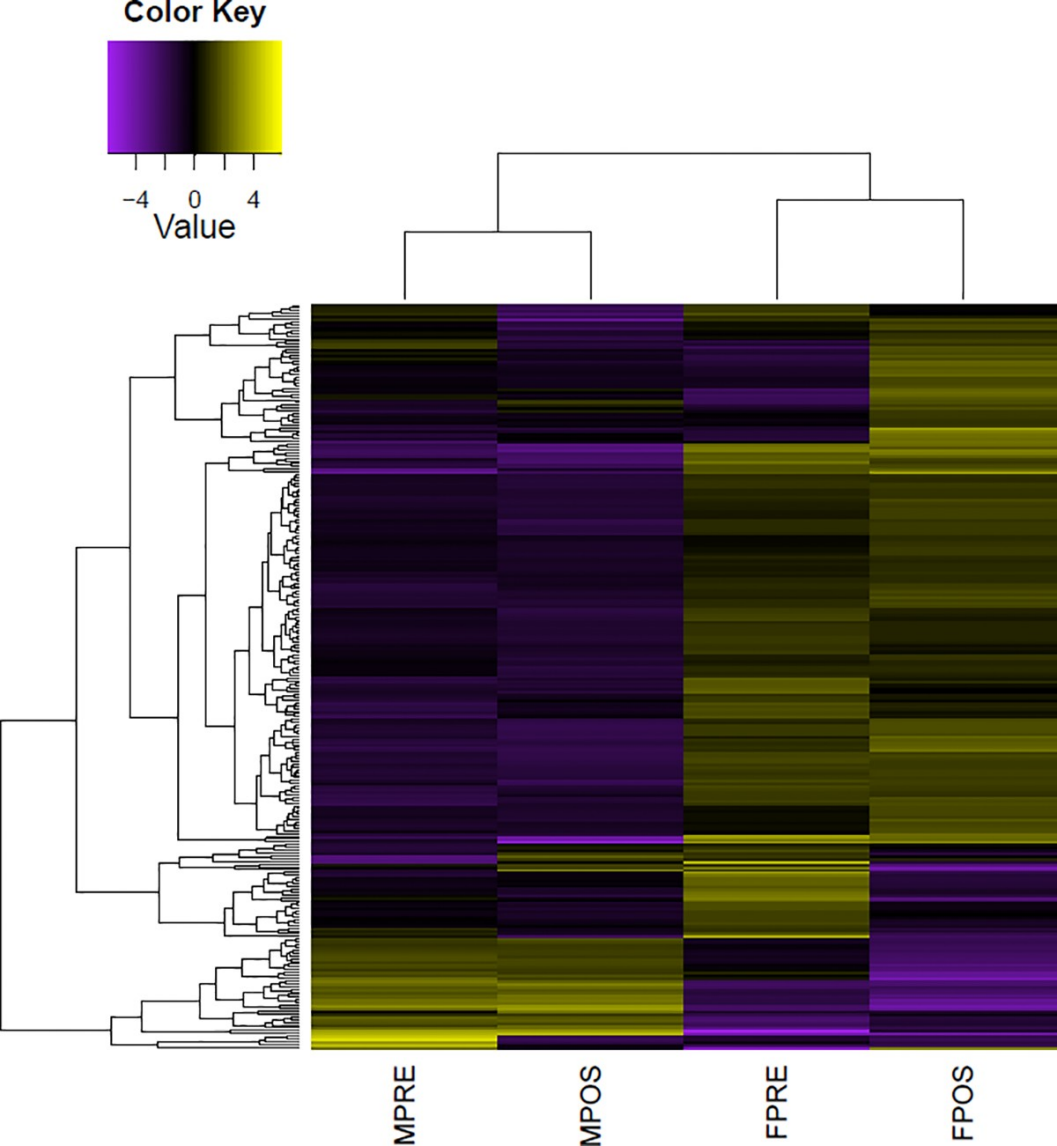
Heatmap representing the abundance of differentially expressed genes (rows, FDR < 0.01, fold change > 2) in each cDNA library (columns). Dendogram shows that female and male *O*. *maya* white body samples were clustered in different groups. Libraries: MPRE = male pre-copula, MPOS = male post-copula, FPRE = female pre-spawning, FPOS = female post-spawning. Values represent fold change in log_2_.

A total of 3,522 WB transcripts showed significant differential expression between females and males (FDR < 0.01). From these, 2,192 showed higher expression in females and 1,330 in males. By contrast, differential gene expression was much lower comparing before and after the reproductive events. Between pre and post-spawning females, only 60 transcripts showed significant differential expression; and in males, 140 transcripts showed significant differences between pre and post copula condition (Fig 1).

### Functional annotation and enrichment analysis

**Fig 2:**
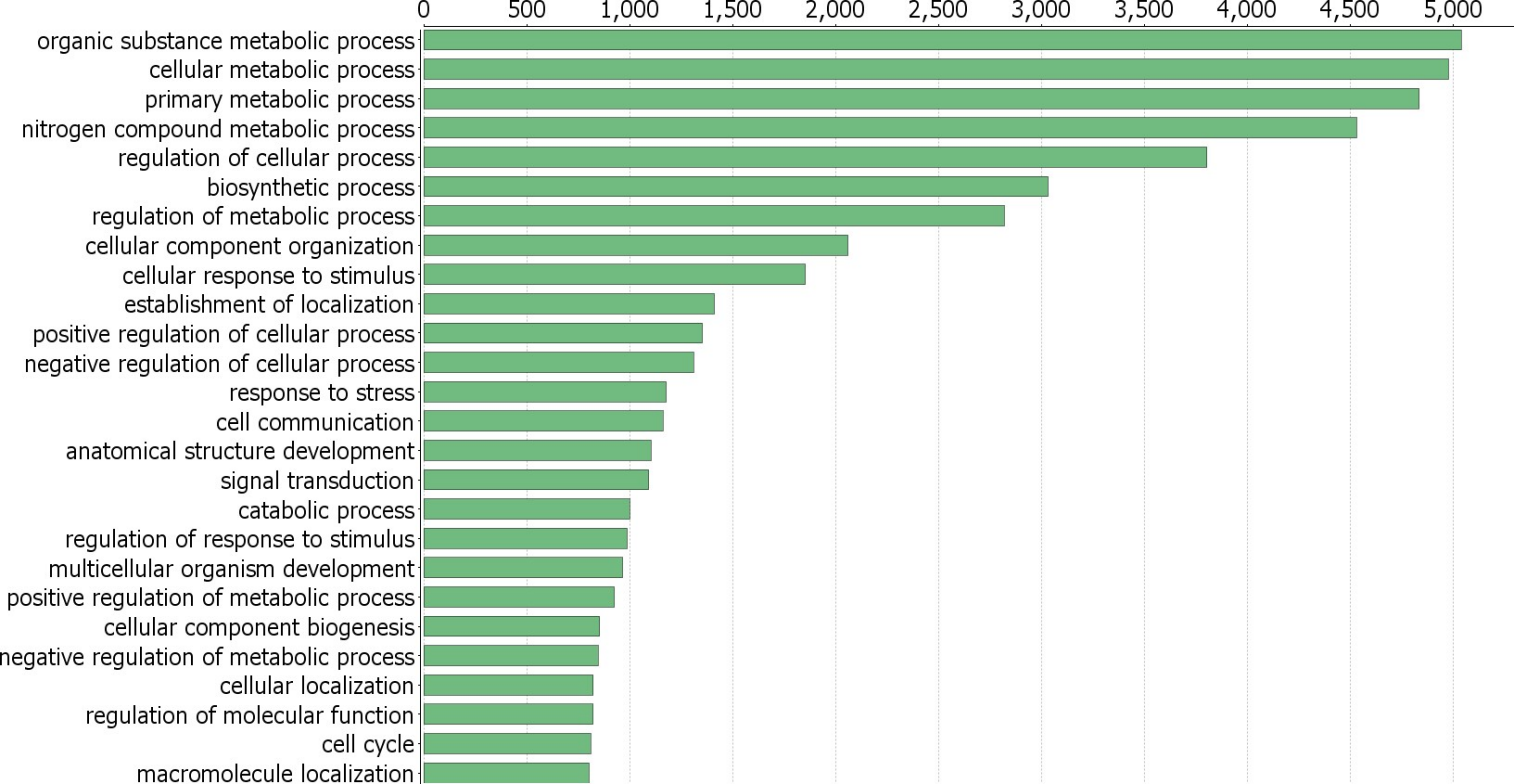
Top GO terms (biological process, level 3) from the reference transcriptome of *O*. *maya* white body. The categories with more transcripts assigned are shown.

Using BLASTx searches, 17,656 transcripts of the reference transcriptome showed significant hits against the UniProt database, corresponding to 10 339 putative peptides. Then, after separating transcripts by sex, 13,399 were annotated in females, corresponding to 9,175 peptides; on the other hand, 13,265 male transcripts corresponding to 9,429 peptides showed significant hits in this database. From these, 5,926 hits were shared, 3,249 were exclusive for females and 3,503 for males. For the reference transcriptome the best represented gene ontologies in terms of biological processes (BP) are shown in Fig 2 where the response to stress, cell communication, signaling, and developmental processes appeared among the most specific categories.

Moreover, in the assembled transcriptome we found at least 74 unigenes from the immune system category (Table 2), as well as 25 additional unigenes coding for different proteins considered as hematopoietic fingerprints [52] (Table 3).

**Table 2:** Transcripts and their encoded putative proteins from *O*. *maya* white body, classified in the immune process GO category.

Transcript ID	Putative encoded protein	E-value
TRINITY_DN3041_c0_g1_i1	Ankyrin repeat and KH domain-containing protein mask	0.00E+00
TRINITY_DN58699_c0_g1_i3	AP-1 complex subunit gamma-1	0.00E+00
TRINITY_DN7437_c0_g1_i1	ATP-binding cassette sub-family F member 3	0.00E+00
TRINITY_DN12624_c0_g1_i1	Canalicular multispecific organic anion transporter 1	0.00E+00
TRINITY_DN13311_c0_g1_i4	Interleukin enhancer-binding factor 2 homolog	0.00E+00
TRINITY_DN25104_c0_g1_i2	Polypeptide N-acetylgalactosaminyltransferase 2	0.00E+00
TRINITY_DN14001_c0_g1_i1	Protein transport protein Sec23A	0.00E+00
TRINITY_DN12936_c0_g2_i1	Protein transport protein Sec24B	0.00E+00
TRINITY_DN57852_c0_g1_i1	Protein transport protein Sec24C	0.00E+00
TRINITY_DN32521_c0_g1_i1	Protein transport protein Sec31A	0.00E+00
TRINITY_DN33465_c0_g1_i1	Ribonuclease 3	0.00E+00
TRINITY_DN16095_c0_g1_i1	Stress-activated protein kinase JNK	0.00E+00
TRINITY_DN58150_c0_g1_i1	Protein pellino	8.16E-172
TRINITY_DN50778_c0_g1_i1	DNA-directed RNA polymerase III subunit RPC5	5.39E-171
TRINITY_DN33525_c0_g1_i1	Cytoplasmic dynein 1 heavy chain 1	9.34E-165
TRINITY_DN2016_c0_g1_i1	AP-1 complex subunit mu-1	2.15E-159
TRINITY_DN25996_c0_g1_i1	DNA-directed RNA polymerase III subunit RPC3	2.69E-149
TRINITY_DN6228_c0_g1_i1	Maspardin	1.44E-146
TRINITY_DN3533_c0_g1_i1	Ras-related protein Rab-14	3.08E-145
TRINITY_DN33699_c0_g1_i1	DNA-directed RNA polymerase III subunit RPC2	1.66E-142
TRINITY_DN1805_c0_g1_i1	DNA-directed RNA polymerase III subunit RPC6	5.03E-137
TRINITY_DN26718_c0_g1_i1	ATP-binding cassette sub-family C member 9	1.67E-134
TRINITY_DN25730_c0_g1_i1	Exosome complex component RRP41	1.66E-123
TRINITY_DN59780_c0_g1_i1	Oxysterol-binding protein-related protein 1	4.42E-122
TRINITY_DN13124_c0_g1_i1	Dynactin subunit 4	1.28E-114
TRINITY_DN17640_c0_g1_i1	85/88 kDa calcium-independent phospholipase A2	1.38E-99
TRINITY_DN1706_c0_g1_i1	Serine/threonine-protein kinase TBK1	4.09E-93
TRINITY_DN14949_c0_g1_i6	Ras-related protein Rab-35	7.03E-91
TRINITY_DN24144_c0_g1_i1	AP-1 complex subunit sigma-2	4.02E-90
TRINITY_DN27946_c0_g1_i1	DNA-directed RNA polymerase III subunit RPC8	9.30E-88
TRINITY_DN12864_c0_g1_i1	Kinesin-like protein KIF22	9.73E-83
TRINITY_DN43844_c0_g1_i1	Ras-related protein Rab-32	5.84E-76
TRINITY_DN12915_c0_g1_i2	Cytosolic carboxypeptidase-like protein 5	8.70E-72
TRINITY_DN12601_c0_g2_i1	Dynactin subunit 6	1.54E-68
TRINITY_DN1393_c0_g1_i1	Exosome complex component RRP46	3.18E-63
TRINITY_DN15204_c0_g1_i2	Histone H2B	3.91E-63
TRINITY_DN30815_c0_g1_i1	Ras-related protein Rab-27A	1.09E-61
TRINITY_DN15285_c2_g1_i6	Death-associated inhibitor of apoptosis 2	2.65E-61
TRINITY_DN9422_c0_g1_i1	DNA-directed RNA polymerase III subunit RPC10	1.49E-58
TRINITY_DN31254_c0_g1_i1	Germinal-center associated nuclear protein	1.64E-54
TRINITY_DN6747_c0_g1_i2	Flavin-containing monooxygenase FMO GS-OX-like 2	7.91E-51
TRINITY_DN43328_c0_g1_i1	Ras-related protein Rab-34	3.11E-48
TRINITY_DN20201_c0_g1_i1	Protein kinase C-like 1	4.07E-40
TRINITY_DN2448_c0_g1_i1	Repressor of yield of DENV protein homolog	2.04E-39
TRINITY_DN16609_c0_g1_i1	DNA-directed RNA polymerase III subunit RPC4	2.34E-39
TRINITY_DN24836_c0_g1_i1	Gamma-interferon-inducible lysosomal thiol reductase	9.97E-39
TRINITY_DN10639_c0_g1_i1	B-cell lymphoma 3 protein	1.25E-38
TRINITY_DN22385_c0_g1_i1	DNA-directed RNA polymerase III subunit RPC1	4.74E-35
TRINITY_DN5407_c0_g1_i1	NF-kappa-B inhibitor cactus	1.36E-34
TRINITY_DN12481_c0_g1_i2	Probable ATP-dependent RNA helicase DHX58	3.54E-34
TRINITY_DN62663_c0_g1_i1	Acyl-CoA-binding protein	4.77E-34
TRINITY_DN18508_c0_g1_i1	S-adenosylmethionine decarboxylase proenzyme	7.69E-32
TRINITY_DN41170_c0_g1_i1	Ecdysone-induced protein 75B, isoform B	2.22E-31
TRINITY_DN51040_c0_g1_i1	Bactericidal permeability-increasing protein	4.77E-31
TRINITY_DN1251_c0_g1_i1	Phosphonopyruvate decarboxylase	1.36E-28
TRINITY_DN14304_c0_g1_i2	Coactosin-like protein	4.35E-28
TRINITY_DN6200_c0_g1_i1	Dicer-like protein 2–1 [Includes: Endoribonuclease dcl2-1	1.26E-26
TRINITY_DN1240_c0_g1_i1	DNA-directed RNA polymerase III subunit RPC9	8.77E-26
TRINITY_DN14178_c0_g1_i1	Serine incorporator 3	9.71E-26
TRINITY_DN65555_c0_g1_i1	Copper-transporting ATPase 1	2.09E-25
TRINITY_DN5713_c0_g2_i1	Somatomedin-B and thrombospondin type-1 domain-containing protein	8.08E-25
TRINITY_DN45827_c0_g1_i1	Zinc finger protein 175	2.29E-22
TRINITY_DN10580_c0_g1_i2	E3 ubiquitin-protein ligase TRIM56	1.24E-18
TRINITY_DN51724_c0_g1_i1	Protein toll	2.59E-16
TRINITY_DN44678_c0_g1_i1	Cathepsin S	1.89E-15
TRINITY_DN18512_c0_g1_i1	Ras-related protein Rab-27A	3.72E-15
TRINITY_DN54582_c0_g1_i1	Indoleamine 2,3-dioxygenase 2	9.08E-13
TRINITY_DN24334_c0_g1_i1	C-type lectin domain family 4 member E	9.92E-13
TRINITY_DN12672_c0_g2_i1	Soluble scavenger receptor cysteine-rich domain-containing protein SSC5D	7.18E-11
TRINITY_DN53340_c0_g1_i2	Putative fungistatic metabolite	1.17E-09
TRINITY_DN14447_c0_g1_i3	B-cell lymphoma 3 protein homolog	7.47E-09
TRINITY_DN50497_c0_g1_i1	Putative phosphatidate phosphatase	7.47E-08
TRINITY_DN11786_c0_g1_i2	Interleukin-1 receptor accessory protein	6.39E-07
TRINITY_DN54047_c0_g1_i1	Venom phosphodiesterase 1	3.94E-06

E-value for the match between query and subject sequences is shown for each transcript.

**Table 3:** Hematopoietic fingerprint proteins [52], detected in the reference transcriptome of *O*. *maya* white body.

Transcript ID	Putative encoded protein	E-value
TRINITY_DN13855_c0_g1_i1	Cis-aconitate decarboxylase (CAD) (EC 4.1.1.6)	1.14E-116
TRINITY_DN18826_c0_g1_i1	E3 ubiquitin-protein ligase FANCL (EC 2.3.2.27)	1.06E-99
TRINITY_DN4253_c0_g1_i1	Zinc finger protein ZFAT	2.43E-81
TRINITY_DN41973_c0_g1_i1	WD repeat-containing protein 78	1.29E-71
TRINITY_DN12997_c0_g1_i2	Serine/arginine-rich splicing factor 4	1.51E-67
TRINITY_DN32443_c0_g1_i1	Armadillo repeat-containing protein 6	1.63E-66
TRINITY_DN5066_c0_g1_i1	Mediator of RNA polymerase II transcription subunit 8	6.43E-63
TRINITY_DN58912_c0_g1_i1	MDS1 and EVI1 complex locus protein EVI1	3.92E-61
TRINITY_DN1806_c0_g1_i1	Protein lunapark	4.69E-57
TRINITY_DN17370_c0_g1_i1	Cytosolic Fe-S cluster assembly factor NARFL	9.31E-39
TRINITY_DN32728_c0_g1_i1	T-lymphoma invasion and metastasis-inducing protein 2 (TIAM-2)	2.77E-33
TRINITY_DN29000_c0_g1_i1	WD repeat-containing protein 38	3.15E-27
TRINITY_DN22647_c0_g1_i1	Prostaglandin G/H synthase 2 (EC 1.14.99.1)	1.63E-25
TRINITY_DN15558_c0_g1_i22	Toll-like receptor 13	6.99E-24
TRINITY_DN19432_c0_g1_i1	Serine-protein kinase ATM	8.17E-24
TRINITY_DN15551_c2_g3_i4	Protein PRRC2C	6.48E-21
TRINITY_DN31382_c0_g1_i1	Zinc finger protein 784	7.85E-17
TRINITY_DN15186_c1_g1_i2	Sorting nexin-8	1.40E-15
TRINITY_DN38869_c0_g1_i1	Collagen alpha-1 (XXIV) chain	2.83E-11
TRINITY_DN26628_c0_g1_i1	Angiopoietin-1 receptor (EC 2.7.10.1)	1.32E-09
TRINITY_DN57286_c0_g1_i1	B-cell lymphoma/leukemia 11A (BCL-11A)	1.69E-09
TRINITY_DN14986_c0_g1_i4	Zinc finger and BTB domain-containing protein 24	3.17E-09
TRINITY_DN40041_c0_g1_i1	WD repeat-containing protein 7	1.82E-08
TRINITY_DN11910_c0_g1_i1	Neural cell adhesion molecule 1-A	3.07E-07
TRINITY_DN54522_c0_g1_i1	Zinc finger homeobox protein 4	2.05E-06

E-value for the match between query and subject sequences is shown for each transcript.

According to the enrichment analysis based on KEGG database, there was high differentiation in signaling pathways between female and male white bodies (Table 4). In the females there was significant enrichment for a wide variety of signaling systems such as Toll-like receptor, PI3K-Atk, TNF, MAPK, mTOR, Rap1, oxytocin, JAK-STAT, VEGF, Ras and insulin among the most important (S1–S13 Figs). By contrast, in males there was enrichment of pathways involving translation initiation factors (eIFs) and apoptotic pathways related to an increment in reactive oxygen species (ROS) (S14–S17 Figs).

**Table 4:** KEGG pathways enriched by the differentially expressed transcripts between female and male WB of *O*. *maya*.

Up in females	Term	P value
	Toll-like receptor signaling pathway	2.05E-04
	Hepatitis B	5.21E-04
	Influenza A	2.36E-03
	Focal adhesion	4.00E-03
	PI3K-Akt signaling pathway	4.00E-03
	Epstein-Barr virus infection	4.65E-03
	Hepatitis C	4.89E-03
	TNF signaling pathway	9.75E-03
	MAPK signaling pathway	1.07E-02
	Chagas disease (American trypanosomiasis)	1.33E-02
	Osteoclast differentiation	1.33E-02
	mTOR signaling pathway	1.49E-02
	Rap1 signaling pathway	1.69E-02
	Bacterial invasion of epithelial cells	2.17E-02
	Shigellosis	2.17E-02
	Thyroid hormone signaling pathway	2.17E-02
	Phagosome	2.78E-02
	Acute myeloid leukemia	3.24E-02
	Jak-STAT signaling pathway	3.24E-02
	Oxytocin signaling pathway	3.24E-02
	Regulation of actin cytoskeleton	3.24E-02
	ErbB signaling pathway	3.61E-02
	Measles	3.61E-02
	Choline metabolism in cancer	4.53E-02
	Fc epsilon RI signaling pathway	4.53E-02
	VEGF signaling pathway	4.53E-02
	Insulin resistance	4.57E-02
	Ras signaling pathway	4.57E-02
**Up in males**		
	Parkinson's disease	1.65E-04
	Oxidative phosphorylation	7.05E-04
	Alzheimer's disease	9.40E-04
	Huntington's disease	5.57E-03
	RNA transport	2.64E-02
	Non-alcoholic fatty liver disease (NAFLD)	3.57E-02
	Pyrimidine metabolism	4.74E-02

P value of the enrichment is shown.

On the other hand, there were also significant GO enrichments based on the transcripts with higher expression in each sex (Table 5). In the females, unigenes involved in the neuropeptide signaling, integrin-mediated signaling, and signaling regulation pathways were highly expressed. Among the transcripts included in the category of neuropeptide signaling, we found five sequences putatively coding for FMRF-amide neuropeptide. These sequences were further analyzed to obtain the predicted coding sequence and the encoded peptide. The longest predicted peptide consisted in 143 amino acids with YIPF repeats each 12 amino acids, but not FMRF repeats were detected. This peptide obtained significant BLASTp hits (1E-10) with the Enterin neuropeptide of the clam *Mizuhopecten yessoensis* (NCBI accession number OWF47724.1). The aligned amino acid sequences presented Y+PF matches each 12 positions with no gaps, and mismatches in the non-repetitive region. This confirms the similarity of these transcripts with other mollusk neuropeptides.

**Table 5:** Top-20 enriched GO (fisher exact test, FDR < 0.05) in female and male *O*. *maya* white body.

Females	FDR
neuropeptide signaling pathway	3.29E-09
protein localization to plasma membrane	2.09E-07
positive regulation of cell migration	4.42E-07
positive regulation of transcription by RNA polymerase II	6.37E-07
protein autophosphorylation	1.11E-06
negative regulation of cell population proliferation	2.76E-06
negative regulation of multicellular organismal process	4.38E-06
cerebral cortex neuron differentiation	2.72E-05
histone H4 acetylation	4.48E-05
protein ubiquitination	5.18E-05
positive regulation of organelle organization	5.74E-05
establishment of endothelial barrier	7.18E-05
embryonic morphogenesis	7.82E-05
primary miRNA processing	1.31E-04
COPII vesicle coating	1.31E-04
regulation of phosphoprotein phosphatase activity	1.31E-04
mRNA splice site selection	1.71E-04
integrin-mediated signaling pathway	1.75E-04
keratinocyte differentiation	2.27E-04
histone H3-K27 trimethylation	2.37E-04
**Males**	
translational initiation	3.73E-08
rRNA processing	9.92E-07
nuclear-transcribed mRNA catabolic process, nonsense-mediated decay	1.45E-05
regulation of translation	1.77E-04
ribonucleoprotein complex assembly	1.94E-04
cytoplasmic translation	2.13E-04
negative regulation of multi-organism process	2.92E-03
proteolysis	4.81E-03
regulation of mRNA splicing, via spliceosome	5.49E-03
dUMP biosynthetic process	5.59E-03
positive regulation of humoral immune response	5.59E-03
proteasome assembly	5.59E-03
ribosomal large subunit biogenesis	5.59E-03
alpha-amino acid biosynthetic process	8.99E-03
regulation of apoptotic process	9.00E-03
coenzyme biosynthetic process	1.45E-02
multicellular organismal process	1.64E-02
viral transcription	1.64E-02
regulation of defense response	2.03E-02
cellular detoxification	2.27E-02

The test-set consisted in the transcripts with significant differential expression. The transcripts from the assembled reference transcriptome were used as reference-set.

In addition, GO terms like stem cell division, response to starvation, response to cortisol and replicative senescence were enriched in females. By contrast, the males showed significant enrichment for GOs like anatomical structure development, microtubule-based process, cilium morphogenesis, protein modification process and regulation of apoptosis among the best-represented categories. The unigenes that best represented the enriched GO categories are shown in Table 6.

**Table 6:** Representative genes for the enriched GO (biological process, BP) in female (F) and male (M) white bodies.

Transcript ID	Putative encoded protein	BP	Expression	E-value
TRINITY_DN12966_c0_g2_i1	CAD protein	RC	FE	0.00E+00
TRINITY_DN7789_c0_g1_i1	Slit homolog 2 protein	RC	FE	2.20E-11
TRINITY_DN41857_c0_g1_i1	Slit homolog 3 protein	RC	FE	1.16E-21
TRINITY_DN58413_c0_g1_i1	Neprilysin	Sen	FE	9.67E-26
TRINITY_DN19432_c0_g1_i1	Serine-protein kinase ATM	Sen	FE	8.17E-24
TRINITY_DN22293_c0_g1_i1	Serine/threonine-protein kinase Chk2	Sen	FE	9.77E-27
TRINITY_DN10693_c0_g1_i1	5'-AMP-activated protein kinase catalytic subunit alpha-2	RS	FU	0.00E+00
TRINITY_DN16292_c0_g1_i1	Death-associated protein 1 (DAP-1)	RS	FU	2.85E-14
TRINITY_DN7555_c0_g1_i1	Myotubularin-related protein 3	RS	FU	0.00E+00
TRINITY_DN2816_c0_g1_i1	Cullin-3	SCD	FU	0.00E+00
TRINITY_DN12835_c0_g1_i1	Dedicator of cytokinesis protein 7	SCD	FU	0.00E+00
TRINITY_DN14659_c0_g1_i4	mRNA decay activator protein ZFP36L2-A	SCD	FU	1.03E-28
TRINITY_DN15394_c0_g1_i7	Contactin-1	Sig	FU	7.86E-11
TRINITY_DN15592_c26_g1_i1	E3 ubiquitin-protein ligase MIB1	Sig	FU	0.00E+00
TRINITY_DN12598_c0_g1_i6	Putative neuropeptide (FMRF-amide like)	Sig	FU	2.70E-10
TRINITY_DN15483_c5_g1_i4	Galanin receptor type 2	Sig	FU	2.39E-11
TRINITY_DN6658_c0_g1_i1	Integrin alpha-4	Sig	FU	3.44E-48
TRINITY_DN13419_c1_g1_i3	Rap guanine nucleotide exchange factor 2	Sig	FU	6.55E-147
TRINITY_DN36930_c0_g1_i1	Bardet-Biedl syndrome 2 protein	AA	ME	2.76E-65
TRINITY_DN27334_c0_g1_i1	Sperm-associated antigen 16 protein	AA	ME	8.87E-69
TRINITY_DN45819_c0_g1_i1	Bardet-Biedl syndrome 4 protein	CM	ME	1.55E-84
TRINITY_DN44314_c0_g1_i1	Cilia- and flagella-associated protein 46	CM	ME	6.63E-15
TRINITY_DN52614_c0_g1_i1	Dynein heavy chain 1, axonemal	CM	ME	0.00E+00
TRINITY_DN5218_c0_g1_i1	Tektin-2	CM	ME	5.31E-121
TRINITY_DN41434_c0_g1_i1	Tektin-3	CM	ME	1.59E-130
TRINITY_DN57504_c0_g1_i1	Geranylgeranyl transferase type-2 subunit beta	AR	MU	0.00E+00
TRINITY_DN29802_c0_g1_i1	Protein disulfide-isomerase A3	AR	MU	5.10E-50
TRINITY_DN4574_c0_g1_i1	Protein DJ-1	AR	MU	4.37E-73
TRINITY_DN15563_c0_g1_i1	TNF receptor-associated factor 2	AR	MU	2.18E-80
TRINITY_DN15548_c1_g1_i1	Tubulin alpha chain	MBP	MU	0.00E+00
TRINITY_DN15548_c1_g1_i10	Tubulin alpha-2/alpha-4 chain	MBP	MU	1.16E-174
TRINITY_DN57742_c0_g1_i1	Tubulin beta chain (Beta-tubulin)	MBP	MU	0.00E+00

E = exclusive, U = upregulated. Processes: response to cortisol (RC), replicative senescence (Sen), response to starvation (RS), stem cell division (SCD), signaling (Sig), axoneme assembly (AA), cilium morphogenesis (CM), apoptosis regulation (AR), microtubule-based process (MBP).

### Phylogenetic analysis of relevant DE transcripts

**Fig 3:**
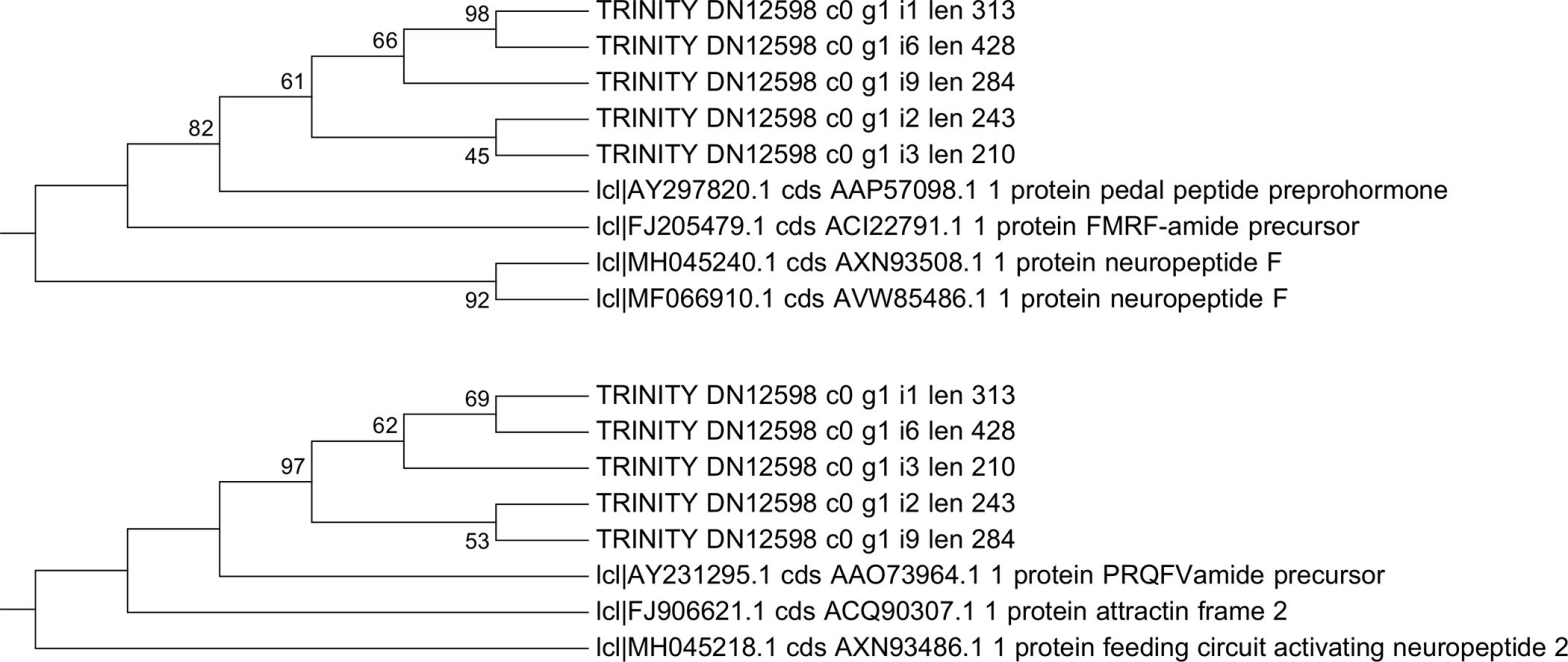
Phylogenetic relationship of the putative neuropeptide-coding transcripts from the *O*. *maya* white body transcriptome. The percentage of replicate trees in which the associated sequences clustered together in the bootstrap test (200 replicates) are shown next to the branches (values above 40% are shown). The analysis involved 90 nucleotide sequences and a total of 255 positions in the final dataset. The subtree including the closer sequences to the putative transcripts are shown. Above: Nucleotide-based analysis. Below: Codon-based analysis.

Phylogenetic analysis for the putative FMRF-amide sequences was performed, including five assembled transcripts. The analysis involved 90 nucleotide sequences and a total of 255 positions in the final dataset. Different topologies were obtained for the codon-based analysis and the nucleotide-based analysis. In the codon-based analysis, the putative FMRF-amide sequences were clustered with *Aplysia californica* PRQFVamide precursor protein mRNA (AY231295.1). However, in the nucleotide-based analysis they were clustered with *Lymnaea stagnalis* pedal peptide preprohormone mRNA (AY297820.1) and *Loligo pealei* FMRF-amide precursor, mRNA (FJ205479.1). Subtrees for both analyses, including the assembled transcripts and their evolutionarily closer sequences are shown in Fig 3.

### Quantitative qPCR analysis

**Fig 4:**
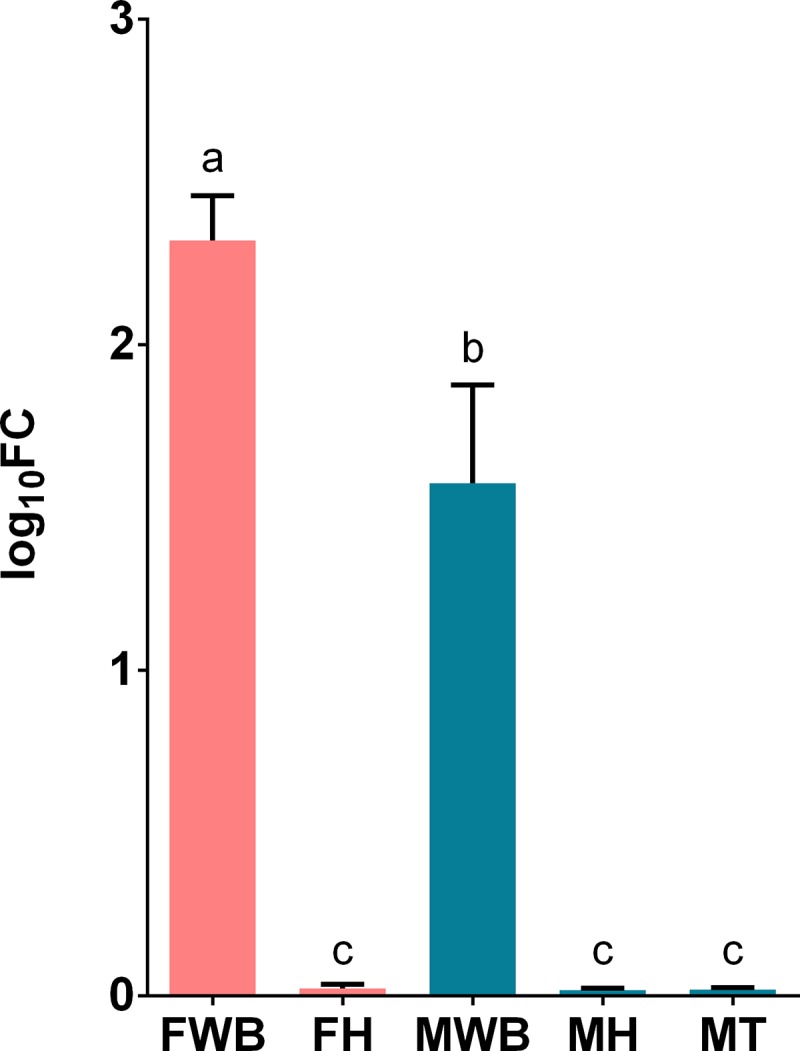
Relative expression levels of the putative neuropeptide transcript TRINITY_DN12598_c0_g1_i6 analyzed via qPCR. Values represent the fold change (log_10_) of each target vs the reference genes. Reference genes: HNRNPD and VATD. Samples: FWB = female white body, FH = female heart, MWB = male white body, MH = male heart, MT = male testis. The 95% confidence interval of each group is shown and the significant differences among groups are represented with different letters.

**Fig 5:**
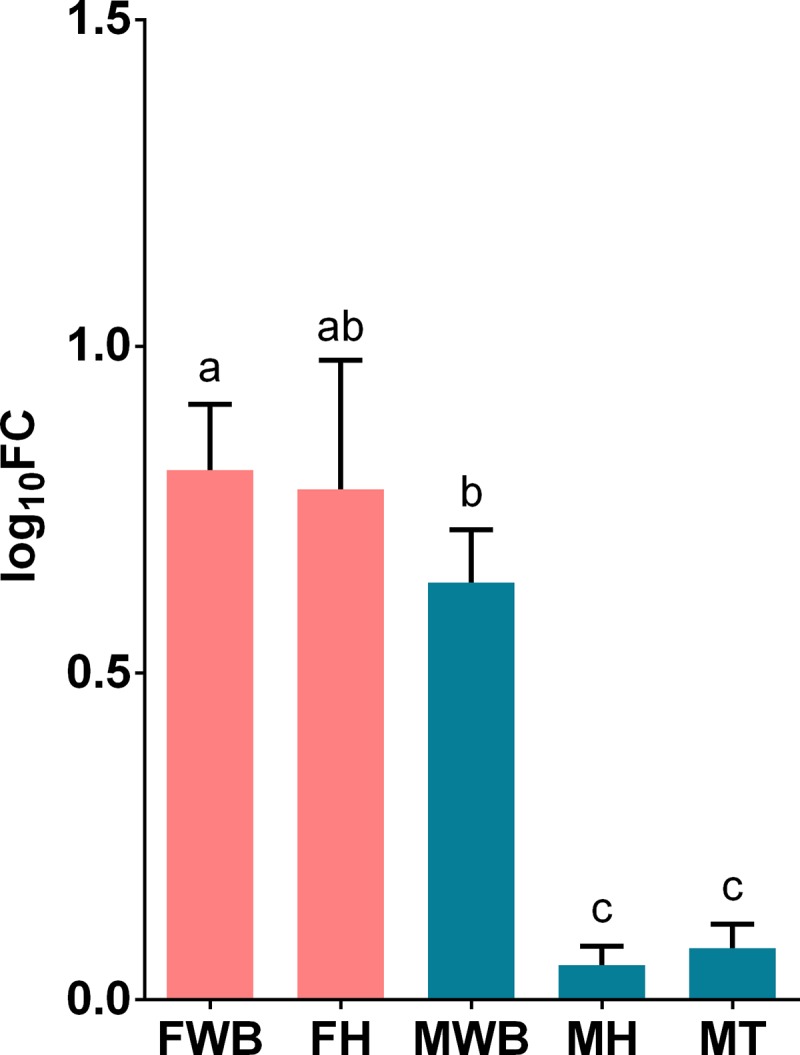
Relative expression levels of corticotropin-releasing hormone receptor 2 (CRHR2) analyzed via qPCR. Values represent the fold change (log_10_) of each target vs the reference genes. Reference genes: HNRNPD and VATD. Samples: FWB = female white body, FH = female heart, MWB = male white body, MH = male heart, MT = male testis. The 95% confidence interval of each group is shown and the significant differences among groups are represented with different letters.

**Fig 6:**
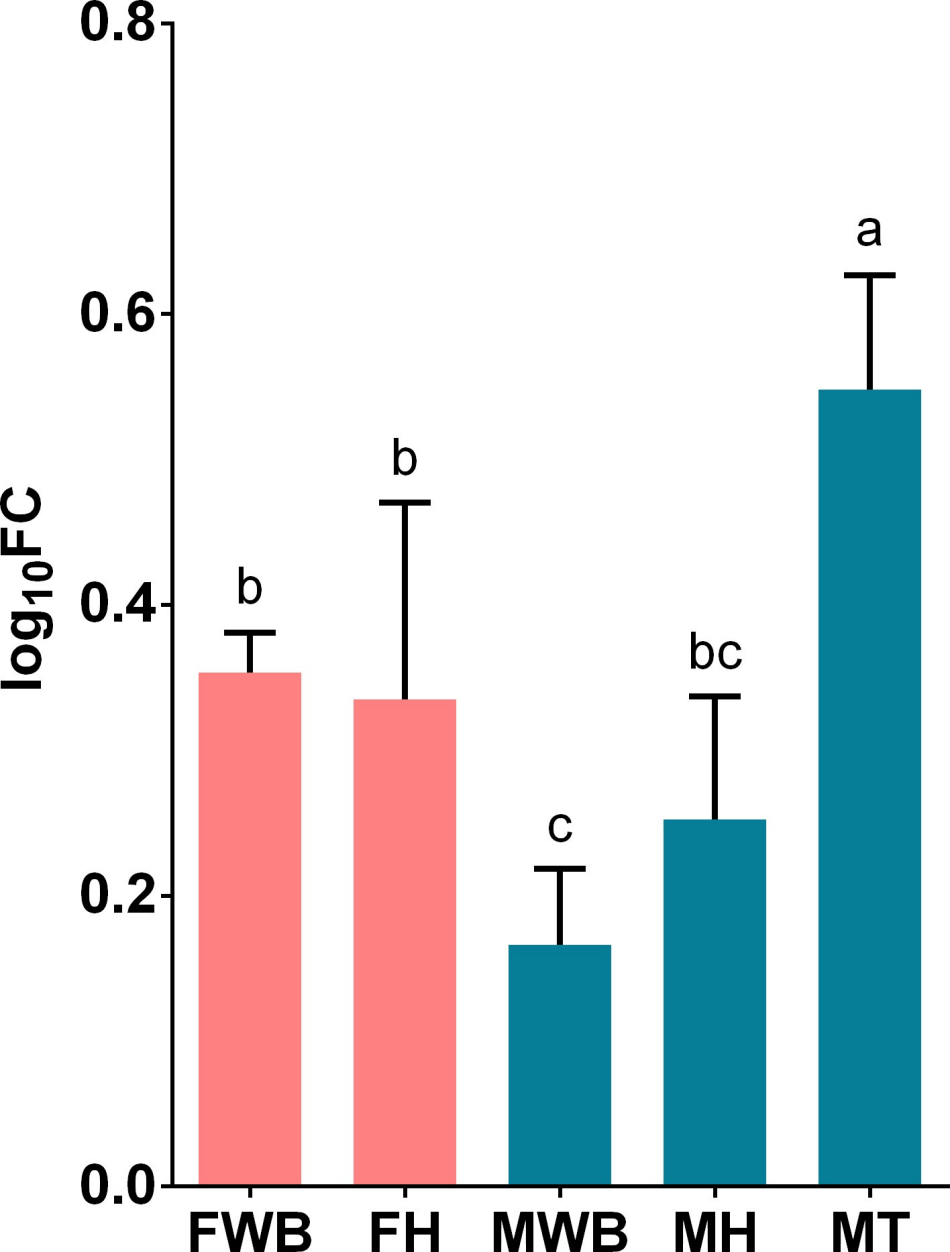
Relative expression levels of estrogen receptor (ESR1) analyzed via qPCR. Values represent the fold change (log_10_) of each target vs the reference genes. Reference genes: HNRNPD and VATD. Samples: FWB = female white body, FH = female heart, MWB = male white body, MH = male heart, MT = male testis. The 95% confidence interval of each group is shown and the significant differences among groups are represented with different letters.

**Fig 7:**
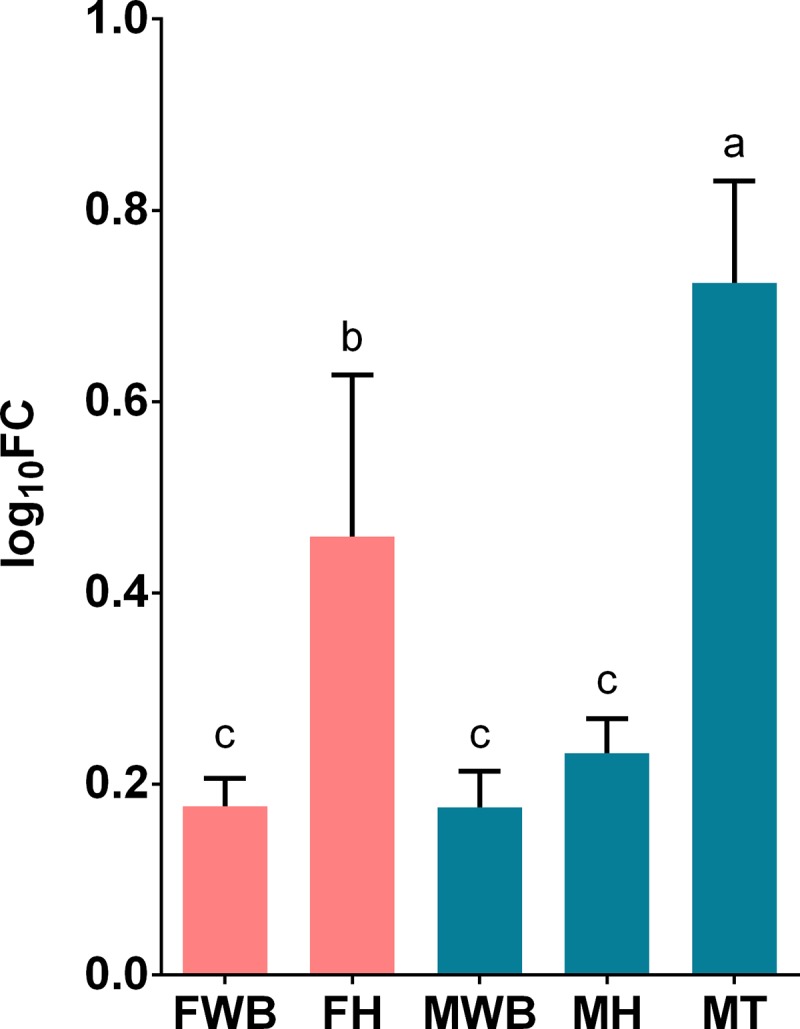
Relative expression levels of estradiol 17-beta-dehydrogenase 8 (HSD17B8) analyzed via qPCR. Values represent the fold change (log_10_) of each target vs the reference genes. Reference genes: HNRNPD and VATD. Samples: FWB = female white body, FH = female heart, MWB = male white body, MH = male heart, MT = male testis. The 95% confidence interval of each group is shown and the significant differences among groups are represented with different letters.

**Fig 8:**
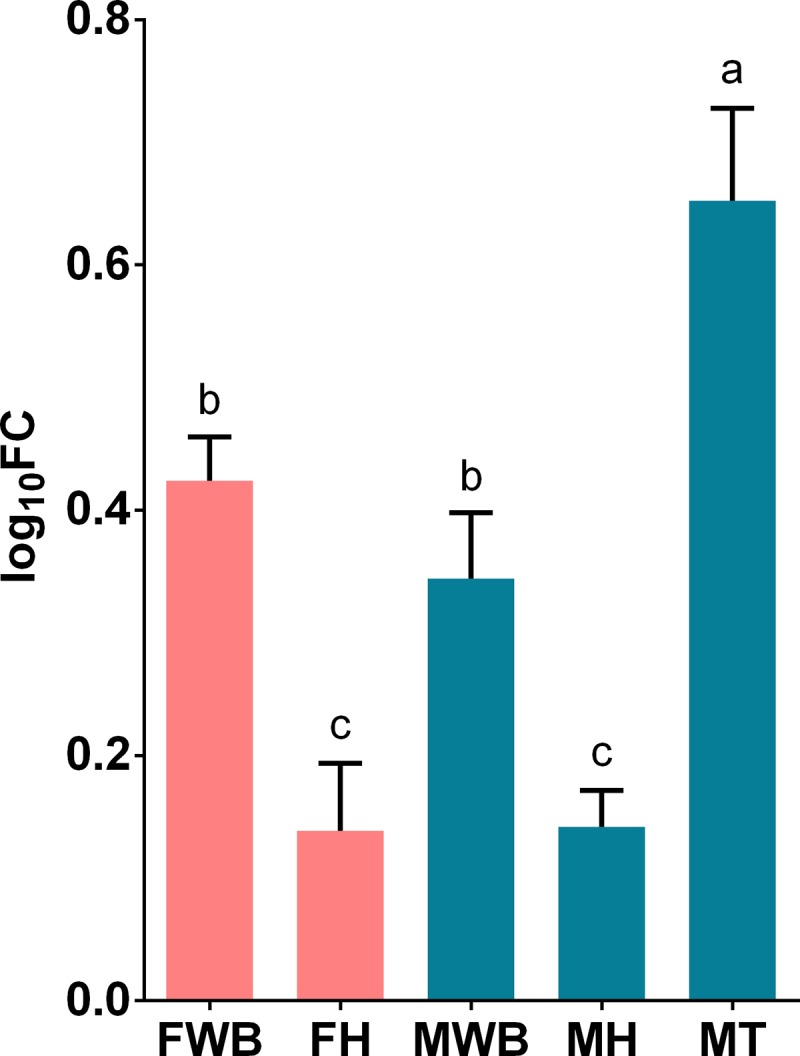
Relative expression levels of ATP-dependent RNA helicase DDX4 (VASA) analyzed via qPCR. Values represent the fold change (log_10_) of each target vs the reference genes. Reference genes: HNRNPD and VATD. Samples: FWB = female white body, FH = female heart, MWB = male white body, MH = male heart, MT = male testis. The 95% confidence interval of each group is shown and the significant differences among groups are represented with different letters.

**Fig 9:**
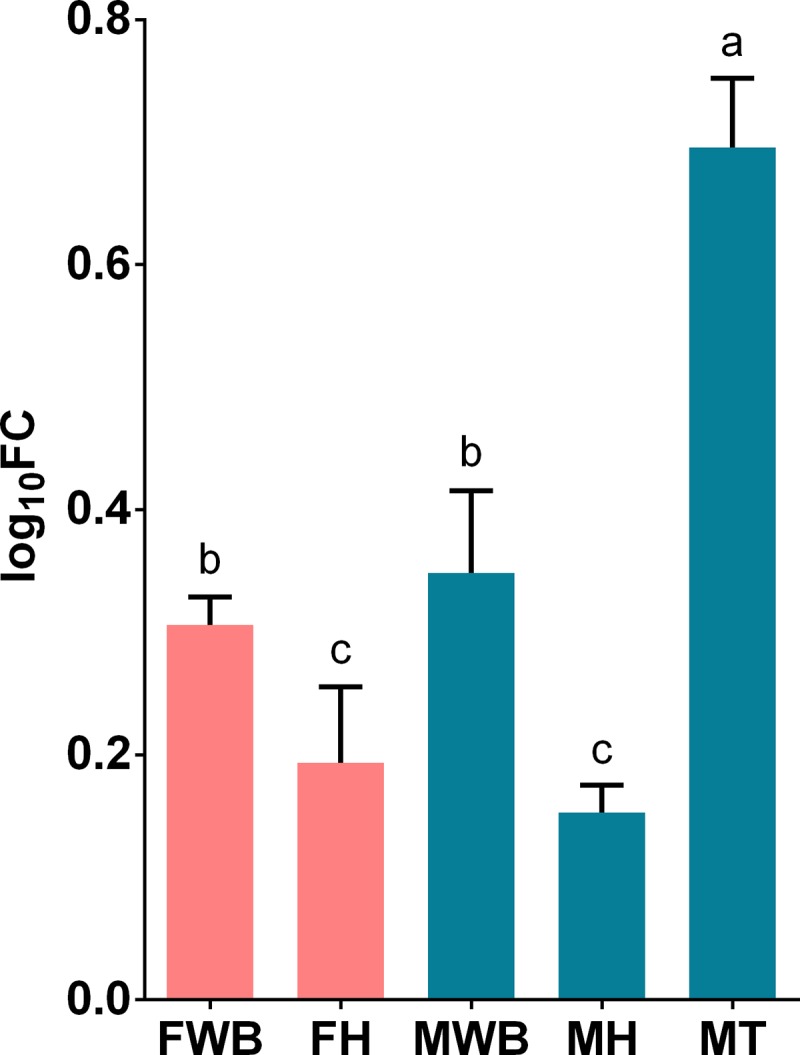
Relative expression levels of piwi-like protein 1 (PIWIL1) analyzed via qPCR. Values represent the fold change (log_10_) of each target vs the reference genes. Reference genes: HNRNPD and VATD. Samples: FWB = female white body, FH = female heart, MWB = male white body, MH = male heart, MT = male testis. The 95% confidence interval of each group is shown and the significant differences among groups are represented with different letters.

**Fig 10:**
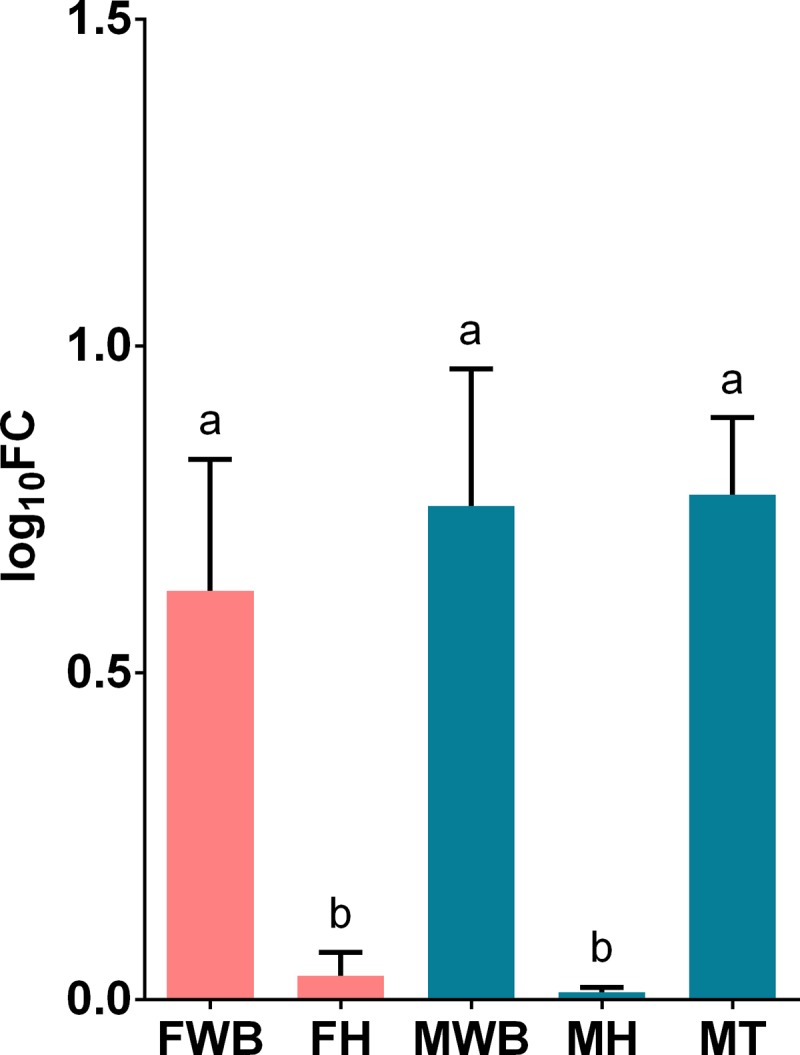
Relative expression levels of zonadhesin (ZAN) analyzed via qPCR. Values represent the fold change (log_10_) of each target vs the reference genes. Reference genes: HNRNPD and VATD. Samples: FWB = female white body, FH = female heart, MWB = male white body, MH = male heart, MT = male testis. The 95% confidence interval of each group is shown and the significant differences among groups are represented with different letters.

**Fig 11:**
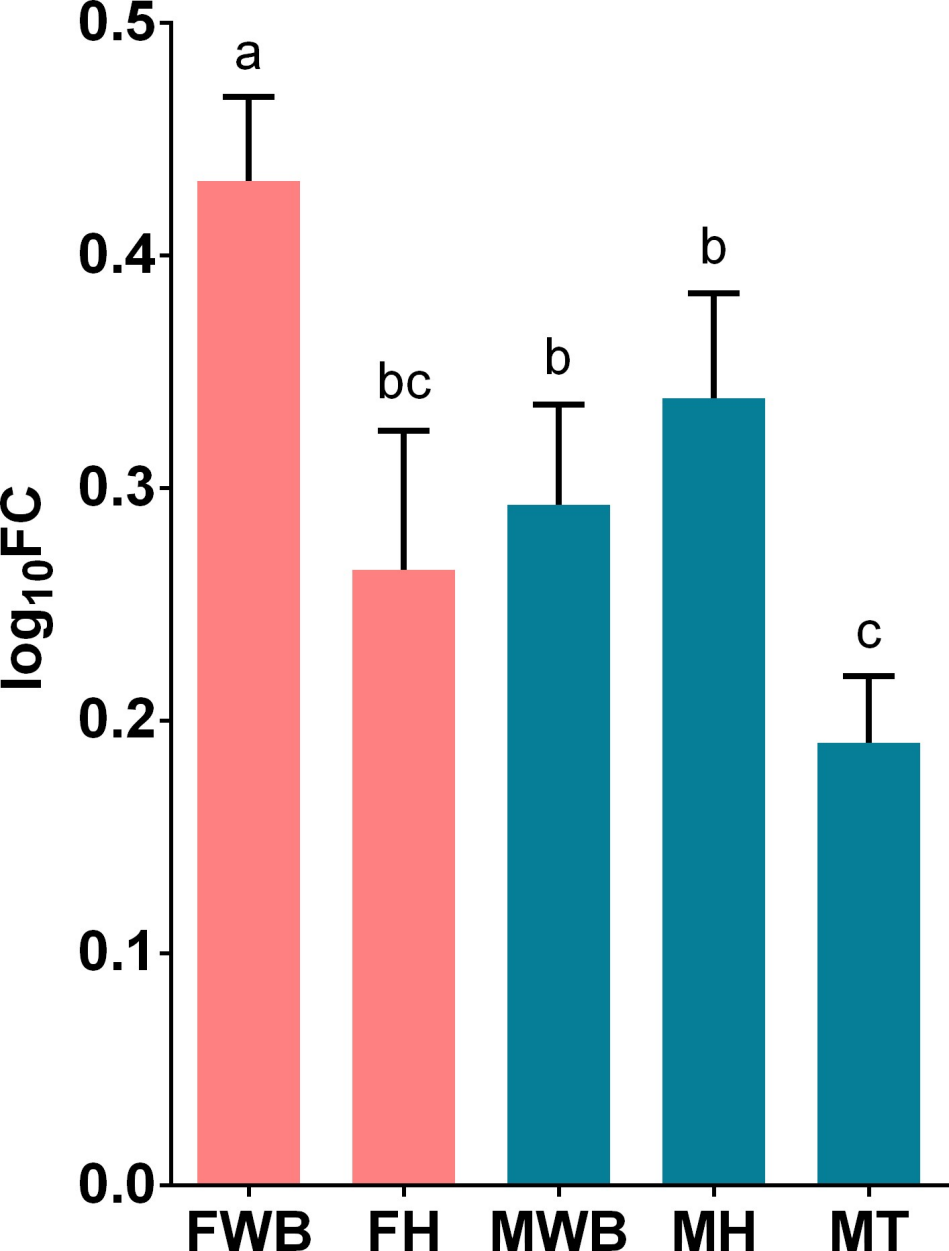
Relative expression levels of C-Jun-amino-terminal kinase-interacting protein 4 (SPAG9) analyzed via qPCR. Values represent the fold change (log_10_) of each target vs the reference genes. Reference genes: HNRNPD and VATD. Samples: FWB = female white body, FH = female heart, MWB = male white body, MH = male heart, MT = male testis. The 95% confidence interval of each group is shown and the significant differences among groups are represented with different letters.

We selected transcripts based on three criteria a) their highly significant differential expression in RNA-seq analysis, like the transcript putatively coding for FMRF-amide neuropeptide, b) their relevance in signaling/hormonal process [53] like estradiol 17-beta-dehydrogenase 8 (HSD17B8), estrogen receptor (ESR1), corticotropin-releasing factor receptor 2 (CRHR2), c) unexpected transcripts that may represent novel functions of the WB like ATP-dependent RNA helicase DDX4 (VASA homolog), Piwi-like protein 1 (PIWIL1) related to germ cell development [54–58], as well as zonadhesin (ZAN) and C-Jun-amino-terminal kinase-interacting protein 4 (SPAG9) transcripts involved in species-specific fertilization [59,60]. All selected transcripts were correctly amplified in real-time qPCR reactions. The expression values of CRHR2, HSD17B and VASA genes failed the Shapiro-Wilk normality test and were analyzed with non-parametric statistics. All transcripts showed significant differential expression among the analyzed groups, considering their parametric or non-parametric distribution (P < 0.01). Their estimated relative expression was represented in histograms with mean values and 95% confidence intervals in each sex-tissue group. Tissue-specific and sex-specific gene expression were detected: the putative FMRF-amide like neuropeptide was detected only in the WB, with higher expression in females than in males (Fig 4); whereas the corticotropin-releasing factor receptor 2, showed higher expression in females’ WB and heart (Fig 5). On the other hand, despite the expression of the estrogen receptor in the WB, the highest expression was observed in the testis tissue; nevertheless, its expression was significantly higher in females’ WB compared to males’ WB (Fig 6). Similarly, HSD17B8 showed relative low expression in WB and the highest expression in testis (Fig 7). In addition, the transcripts related to germ cell development and spermatogenesis detected in the WB like the ATP-dependent RNA helicase DDX4 (VASA) and Piwi-like protein 1 (PIWIL1) were not tissue-specific and showed higher expression in testis (Figs 8 and 9). However, ZAN that is involved in species-specific fertilization, was highly expressed in WB, showing similar expression levels to those in testis (Fig 10). Finally, SPAG9, which is also involved in fertilization showed the highest expression in the females’ WB (Fig 11). The Spearman correlation for the expression values obtained by RNA-Seq and qPCR was significant (P < 0.05), suggesting that both methods reported same tendencies in the expression levels across all the analyzed genes and samples.

## Discussion

One of the most relevant findings in this study was the different transcription pattern between male and female white bodies of *O*. *maya*, during the reproductive phase. The unigenes involved in this difference, fall mainly in the category of signaling pathways, and showed higher expression in females. Numerous signaling cascades were highly active and occurring simultaneously in the females. The enriched KEGG pathways suggest that bacterial and viral infections were taking place in females (Table 4), triggering multiple signaling systems.

At the same time, the neuropeptide signaling pathway was also enriched in females by the high expression of at least 5 different transcripts coding for a putative FMRF-amide like neuropeptide. FMRF-amide related peptides (FaRPs) constitute an evolutionary conserved and diverse group of neuropeptides in the central nervous system (CNS) of many metazoans [61]. Among the functions of FMRF-amide and FaRPs in mollusks are the following: the modulation of sensory organs, reproduction, motility, osmoregulation, feeding and neurogenesis [62,63]. In *O*. *vulgaris*, Di Cosmo and Di Cristo [64], and Di Cristo et al. [65], demonstrated the presence of FMRF-amide both in the CNS and peripheral nervous system (PNS). In the CNS this neuropeptide is involved in the inhibition of the secretory activity of the optic gland, which in turn controls the gonad maturation [64]. FMRF-amide has been detected in several lobes such as optic, subpedunculate and olfactory lobes of *O*. *vulgaris* CNS [66]. Significant hits with this neuropeptide using the UniProt database were obtained in this study. However, when the transcripts sequences were phylogenetically analyzed including all reported molluscan neuropeptides within the NCBI nucleotide database, they were clustered in different ways, depending on the sequence alignment method. In the codon-based analysis the putative neuropeptide transcripts were clustered with *Aplysia californica* PRQFVamide precursor protein mRNA, which is involved in the regulation of the feeding system [67]. By contrast, in the nucleotide-based analysis, the same transcripts were clustered with *Lymnaea stagnalis* pedal peptide preprohormone mRNA, which was highly expressed during parasitic infections in the great pond snail [68]; this branch also included the *Loligo pealei* FMRF-amide precursor, mRNA (FJ205479.1). Despite certain similarity at the transcript level with the FMRF-amide sequence, our query sequences do not contain FMRF repeats, instead they present YIPF repeats each 12 aa according to the longest ORF predicted. This 12-aa distance between repeats is also observed in the Enterin neuropeptide of the clam *M*. *yessoensis*, which may play a role in non-feeding behaviors [69]. This suggests that these tissue-specific transcripts with higher expression in females, show a neuropeptide-like structure, especially due to the constant and equidistant YIPF repeats. This is the first report of putative neuropeptide-like transcripts detected in the octopus’ WB. In this regard, further analyses are needed to clarify the nature and functions of these putative neuropeptide transcripts, to assign it a possible role in the anorexic behavior observed in fertilized females. Furthermore, the response to starvation was another gene ontology enriched in females. This response may be related to the egg protecting behavior during pre and post spawning phases [70,71]. As other incirrate octopods, *O*. *maya* females decrease and stop feeding during incubation of the eggs and focus exclusively on their care until the hatching of the juveniles [70]. Maternal care generally includes the protection of the egg mass from potential predators, ventilation by flushing water through the eggs, cleaning the surface of the eggs, and removing dead embryos [72]. These results suggest that WB may contribute to the regulation of this anorexic behavior in adult *O*. *maya* females.

Similarly, the response to cortisol was another enriched GO in females, which may be linked to their anorexic behavior. Food deprivation has been correlated with high levels of cortisol in fishes [73–75]. A similar glucocorticoid analogous to cortisol, the corticosterone has been detected in *Enteroctopus dofleini* feces after stressful events [76]; so, it is possible that this molecule is released in response to stress conditions [12] also in *O*. *maya* with higher expression in females due to its anorexic behavior before spawning. The presence in the WB of the gene encoding for corticosteroid 11-beta-dehydrogenase isozyme 2 (HSD11B2), which participates in the corticosterone inactivation [77,78], together with the corticotropin-releasing factor receptor 2 (CRHR2), which expression was higher in females, supports this hypothesis. These results suggest that the WB plays a role in the glucocorticoid metabolism, and in the response to glucocorticoids. In this regard, the WB can be an important target of glucocorticoids, considering that these molecules can restrain the immune and inflammatory responses [79–83], which are mediated by the WB at least partially [8,84]. But why the immune response should be down-regulated? In fertilized females this regulation could be required to permit the maintenance of foreign cells, specifically the spermatozoa, which are stored in the females’ oviductal gland up to four months [70,85–87]. The immune regulation leaded by glucocorticoids with possible action in the WB, may contribute to keep spermatozoa safe from the females’ immune system. This down-regulation over females’ immune response may be linked to the high expression of signaling genes involved in bacterial and viral infections, as illustrated by the enriched KEEG pathways. On the other hand, despite some evidences of steroids hormones pathways in the nervous system and gonads in *O*. *vulgaris* [88,89], the precise gland or nervous lobe were the octopus’ glucocorticoids are synthesized remain unclear.

In contrast to females, different processes were enriched by the highly expressed transcripts in males’ WB. Transcripts from the androgen signaling pathway, encoding proteins such as nuclear receptor activator 4, prohibitin and protein DJ-1, were significantly more abundant in males. This result together with the higher expression of the estrogen receptor in females, support the idea that steroid hormones are involved in female and male physiological dimorphism during reproduction [89].

Regarding the enriched KEGG pathways, apparently the males suffered a mitochondrial dysfunction, resulting in higher production of reactive oxygen species (ROS), in a similar way to that observed in the Huntington disease pathway (S16 Fig). Moreover, ROS metabolism was also enriched in males by the high expression of transcripts encoding peroxiredoxin-1, peroxiredoxin-4, superoxide dismutase, autophagy protein 5 and NADH dehydrogenase [ubiquinone] iron-sulfur protein 3. Notably, this apparent mitochondrial dysfunction leading to high ROS production and subsequent induction of the antioxidant system, could be an adaptation for the defense against pathogens. This system was also well represented in the transcriptome of *O*. *vulgaris* hemocytes [19], which in turn are originated in the WB [10]. Authors have suggested that the antioxidant system enzymes may play a role in the defense against pathogens by the hemocytes [19,90,91]. On the other hand, the apoptotic process was also conspicuous in the *O*. *vulgaris* hemocyte transcriptome, with high expression of initiator and effector proteins for apoptosis after parasitic infection [19]. This mechanism is also a major defense against pathogens [92]. In this study, the regulation of apoptosis was an enriched process in males’ WB by the high expression of transcripts coding for TNF receptor-associated factor 2, apoptotic chromatin condensation inducer in the nucleus, Bcl-2 homologous antagonist/killer (apoptosis regulator BAK), geranylgeranyl transferase type-2 subunit beta, RNA-binding protein 5, ribosomal L1 domain-containing protein 1, and 40S ribosomal protein S3, which were classified as apoptotic inducers according to the GO. In this regard, the higher expression of antioxidant enzymes and the positive regulation of apoptosis in males’ WB could be linked to a higher production of hemocytes compared to females.

In the case of unigenes detected exclusively in males, they were classified mainly in microtubule-based process and cilium morphogenesis, despite there is no evidence so far of ciliated cells within the WB [4,10,11]. However, this organ is tightly connected to nervous tissue, especially to optic tracts and optic lobes, where microtubule-based processes are essential during neurogenesis, playing a role in the organization and dynamics of axons and dendrites [93]. In this regard, the WB may be involved in neurogenesis, possibly due to its high stem cell content, and its proximity to nervous tissue. Recently, Bertapelle et al. [94] detected neurogenesis in the CNS of adult *O*. *vulgaris*, which is the first report of adult neurogenesis in lophotrochozoan animals. By contrast, these cilium-related unigenes were not detected in the females’ WB. This can be due to a reduction in neurogenesis because of a more advanced senescence in females compared to males.

Finally, in males’ WB there were enrichments for spermatogenesis-related GO terms, however this could be a coincidence causing an incorrect GO assignment, considering that the molecular basis of the stem cell system of hematopoiesis and spermatogenesis appears to be very similar [95,96]. This incorrect GO assignment was confirmed by the qPCR results, where the expression levels of transcripts involved in spermatogenesis (VASA homolog and PIWIL1) were compared to those in testis. We observed that despite their presence in the white body, their expression was significantly higher in testis. However, ZAN and SPAG9, which are involved in sperm-egg interaction [59,60], were highly expressed in both female and male white bodies, ZAN showing similar levels to those detected in testis and SPAG9 even higher than testis. These proteins could be synthesized by independent tissues (e.g. WB and gonads) to enhance fertilization, but further research is needed to test this hypothesis.

## Conclusion

The results obtained in this study, are evidences of the involvement of *O*. *maya* WB in hematopoiesis and in the regulation of immune processes. Notably, there was an important differentiation of signaling pathways between female and male white bodies. Multiple signaling cascades were upregulated in females, some of them related to bacterial and viral infections. At the same time, females showed higher expression of unigenes related to neuropeptide signaling pathways, as well as unigenes involved in the response to glucocorticoids and starvation. By contrast, in males we detected higher expression of unigenes required in the androgen signaling pathway, antioxidant response, and apoptosis. Considering that glucocorticoids can suppress immunity and the antioxidant/apoptotic response can enhance the defense against pathogens; we can infer that immune response was down-regulated in fertilized females and up-regulated in mature males, which is congruent with the higher gene expression related to infection pathways detected in females. Furthermore, our data suggest for the first time, an involvement of this organ in the physiological differences between mature males and females, showing differential gene expression processes in a sex-specific way during the reproductive phase.

## Supporting information

S1 Fig PI3K-AKT signaling pathway. This pathway was enriched in *O*. *maya* female WB, the red star symbols indicate the proteins encoded by the upregulated unigenes in females.
(TIF)

S2 Fig Focal adhesion. This pathway was enriched in *O*. *maya* female WB, the red star symbols indicate the proteins encoded by the upregulated unigenes in females.
(TIF)

S3 Fig Toll-like receptor signaling pathway. This pathway was enriched in *O*. *maya* female WB, the red star symbols indicate the proteins encoded by the upregulated unigenes in females.
(TIF)

S4 Fig MAPK signaling pathway. This pathway was enriched in *O*. *maya* female WB, the red star symbols indicate the proteins encoded by the upregulated unigenes in females.
(TIF)

S5 Fig RAP1 signaling pathway. This pathway was enriched in *O*. *maya* female WB, the red star symbols indicate the proteins encoded by the upregulated unigenes in females.
(TIF)

S6 Fig TNF signaling pathway. This pathway was enriched in *O*. *maya* female WB, the red star symbols indicate the proteins encoded by the upregulated unigenes in females.
(TIF)

S7 Fig ERBB signaling pathway. This pathway was enriched in *O*. *maya* female WB, the red star symbols indicate the proteins encoded by the upregulated unigenes in females.
(TIF)

S8 Fig RAS signaling pathway. This pathway was enriched in *O*. *maya* female WB, the red star symbols indicate the proteins encoded by the upregulated unigenes in females.
(TIF)

S9 Fig Thyroid hormone signaling pathway. This pathway was enriched in *O*. *maya* female WB, the red star symbols indicate the proteins encoded by the upregulated unigenes in females.
(TIF)

S10 Fig Oxytocin signaling pathway. This pathway was enriched in *O*. *maya* female WB, the red star symbols indicate the proteins encoded by the upregulated unigenes in females.
(TIF)

S11 Fig JAK-STAT signaling pathway. This pathway was enriched in *O*. *maya* female WB, the red star symbols indicate the proteins encoded by the upregulated unigenes in females.
(TIF)

S12 Fig VEGF signaling pathway. This pathway was enriched in *O*. *maya* female WB, the red star symbols indicate the proteins encoded by the upregulated unigenes in females.
(TIF)

S13 Fig Bacterial invasion of epithelial cells. This pathway was enriched in *O*. *maya* female WB, the red star symbols indicate the proteins encoded by the upregulated unigenes in females.
(TIF)

S14 Fig Parkinson disease. This pathway was enriched in *O*. *maya* male WB, the red star symbols indicate the proteins encoded by the upregulated unigenes in males.
(TIF)

S15 Fig Oxidative phosphorylation. This pathway was enriched in *O*. *maya* male WB, the red star symbols indicate the proteins encoded by the upregulated unigenes in males.
(TIF)

S16 Fig Huntington disease. This pathway was enriched in *O*. *maya* male WB, the red star symbols indicate the proteins encoded by the upregulated unigenes in males.
(TIF)

S17 Fig RNA transport. This pathway was enriched in *O*. *maya* male WB, the red star symbols indicate the proteins encoded by the upregulated unigenes in males.
(TIF)
