# Supplementary figures and images for: Octopus maya white body show sex-specific transcriptomic profiles during the reproductive phase, with high differentiation in signaling pathways
Source: PLoS One. 2019 May 16;14(5):e0216982. doi: 10.1371/journal.pone.0216982 (PMC6522055; doi:10.1371/journal.pone.0216982)

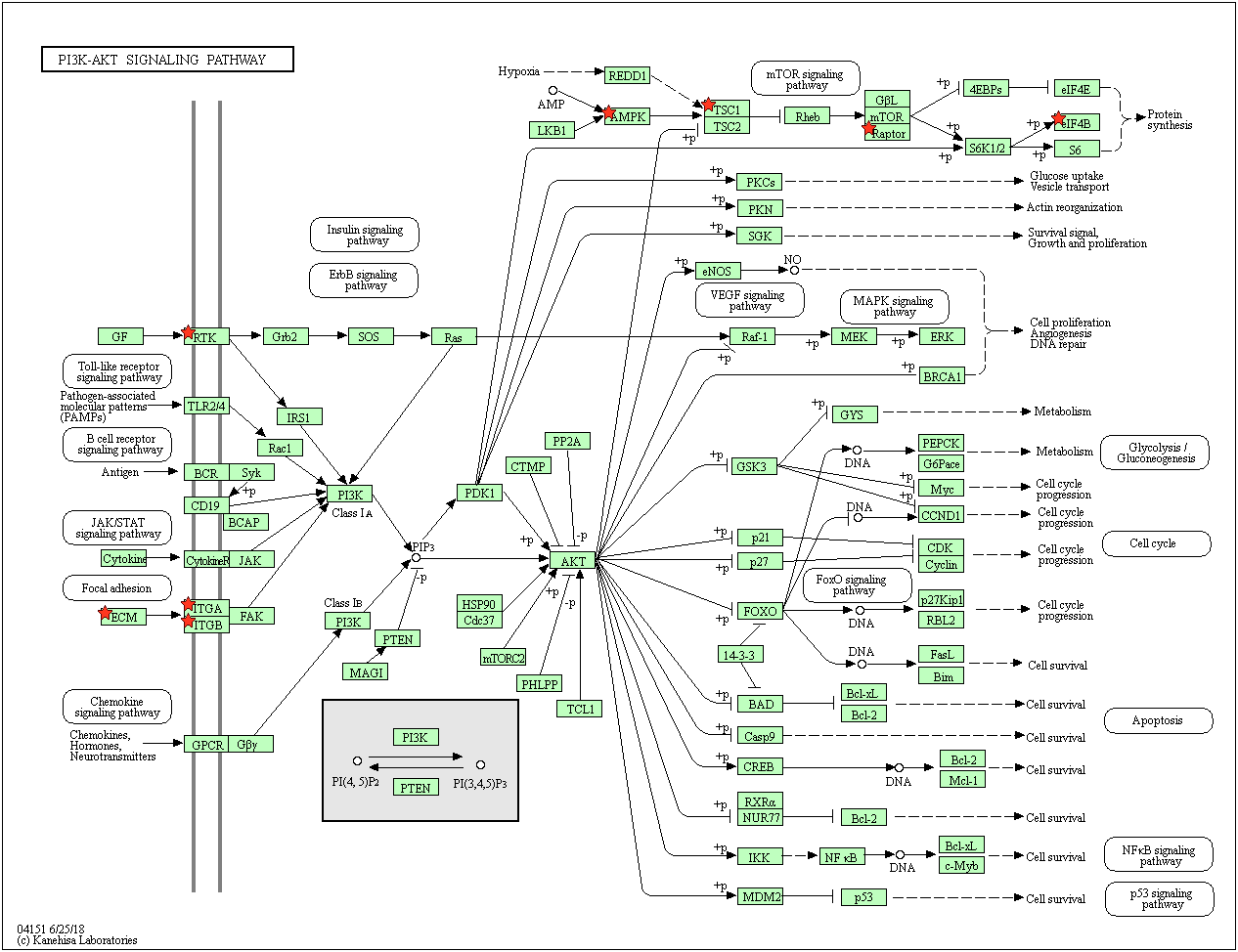

Supplement: S1 Fig — This pathway was enriched in O. maya female WB, the red star symbols indicate the proteins encoded by the upregulated unigenes in females. (TIF) [file pone.0216982.s001.tif]

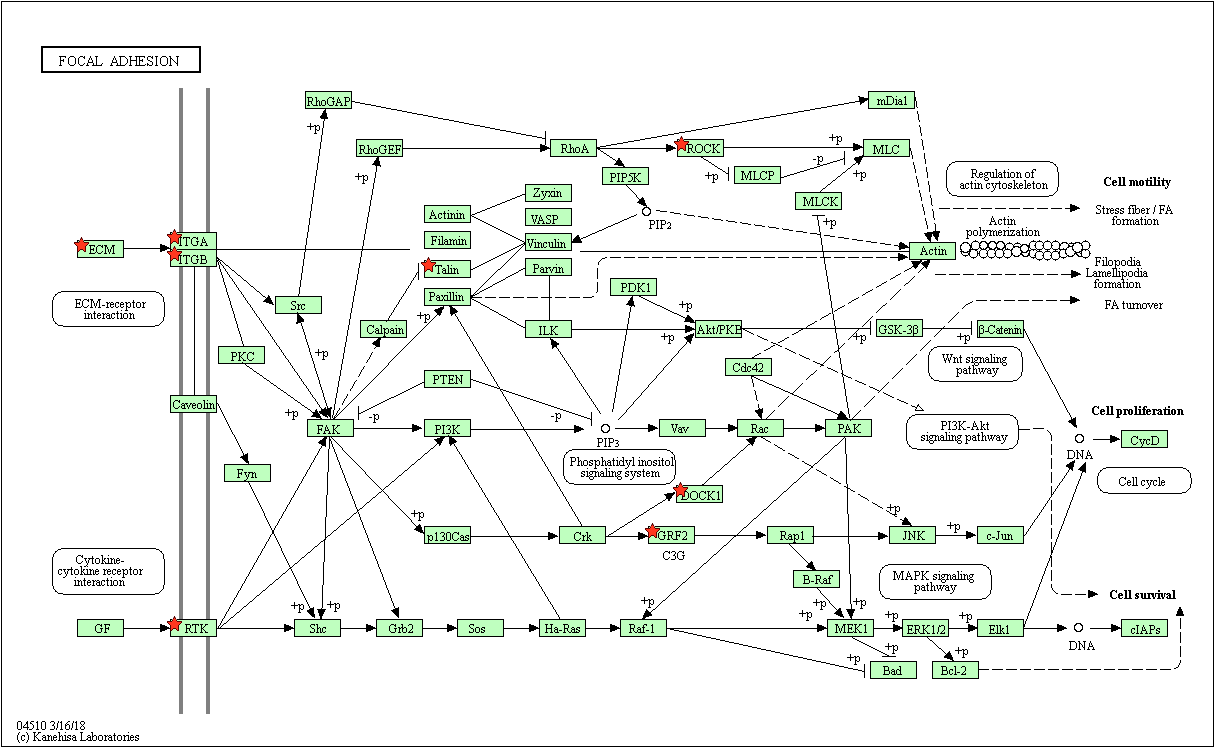

Supplement: S2 Fig — This pathway was enriched in O. maya female WB, the red star symbols indicate the proteins encoded by the upregulated unigenes in females. (TIF) [file pone.0216982.s002.tif]

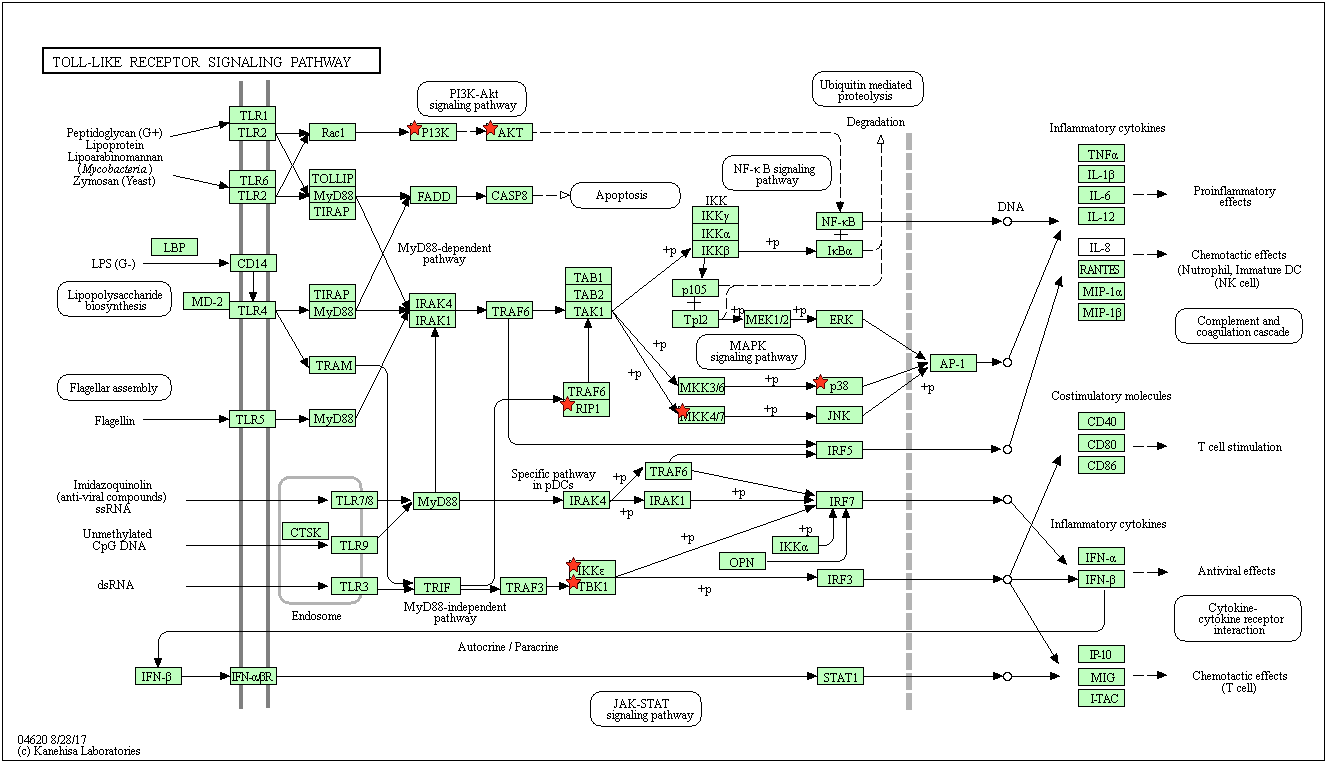

Supplement: S3 Fig — This pathway was enriched in O. maya female WB, the red star symbols indicate the proteins encoded by the upregulated unigenes in females. (TIF) [file pone.0216982.s003.tif]

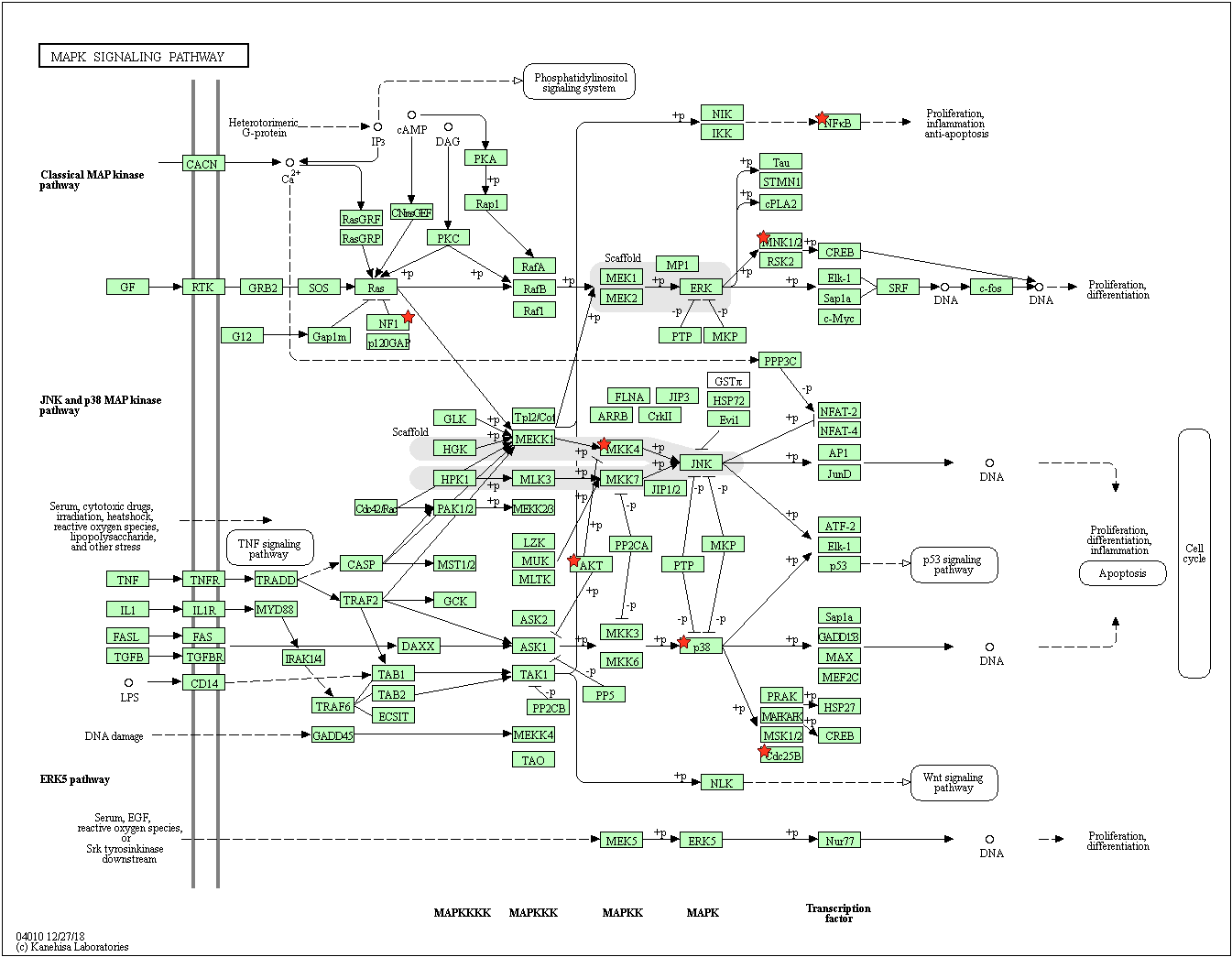

Supplement: S4 Fig — This pathway was enriched in O. maya female WB, the red star symbols indicate the proteins encoded by the upregulated unigenes in females. (TIF) [file pone.0216982.s004.tif]

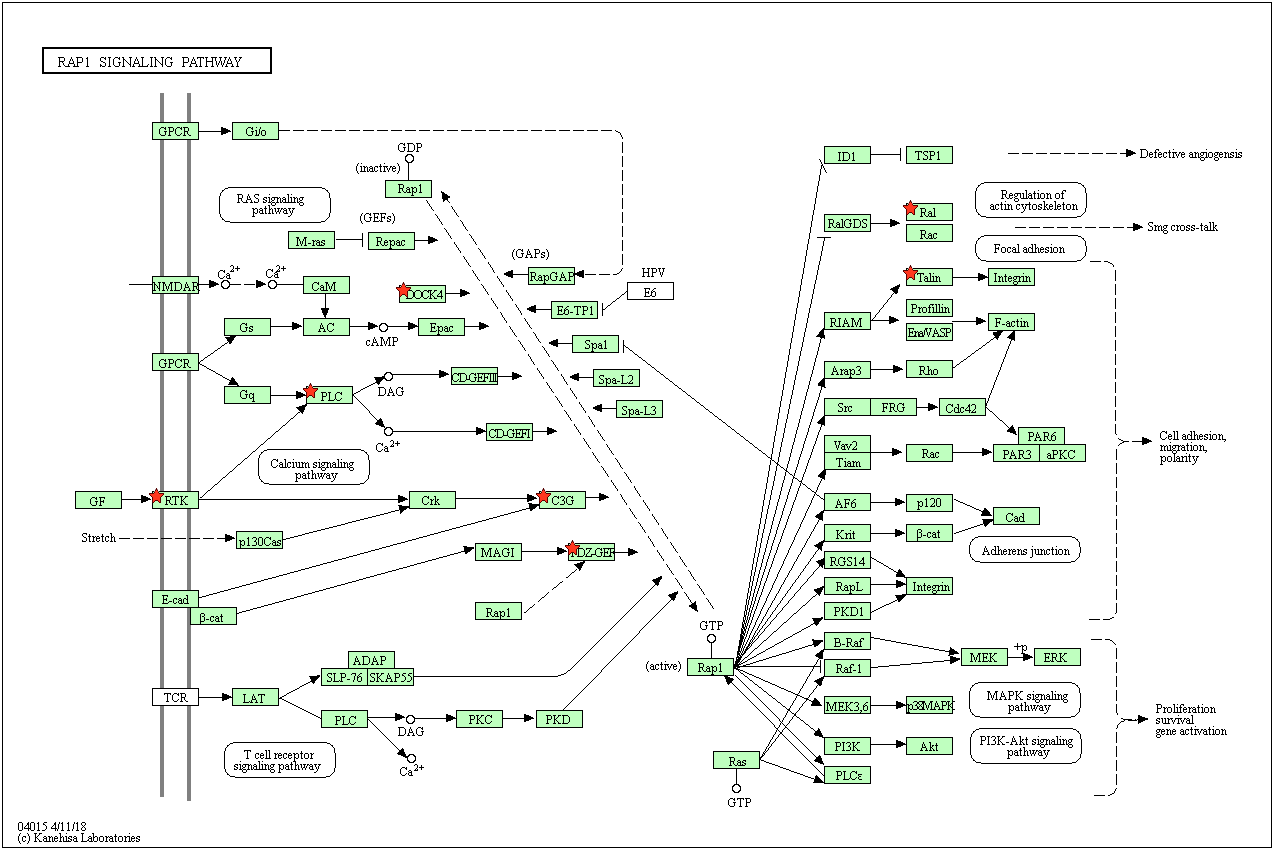

Supplement: S5 Fig — This pathway was enriched in O. maya female WB, the red star symbols indicate the proteins encoded by the upregulated unigenes in females. (TIF) [file pone.0216982.s005.tif]

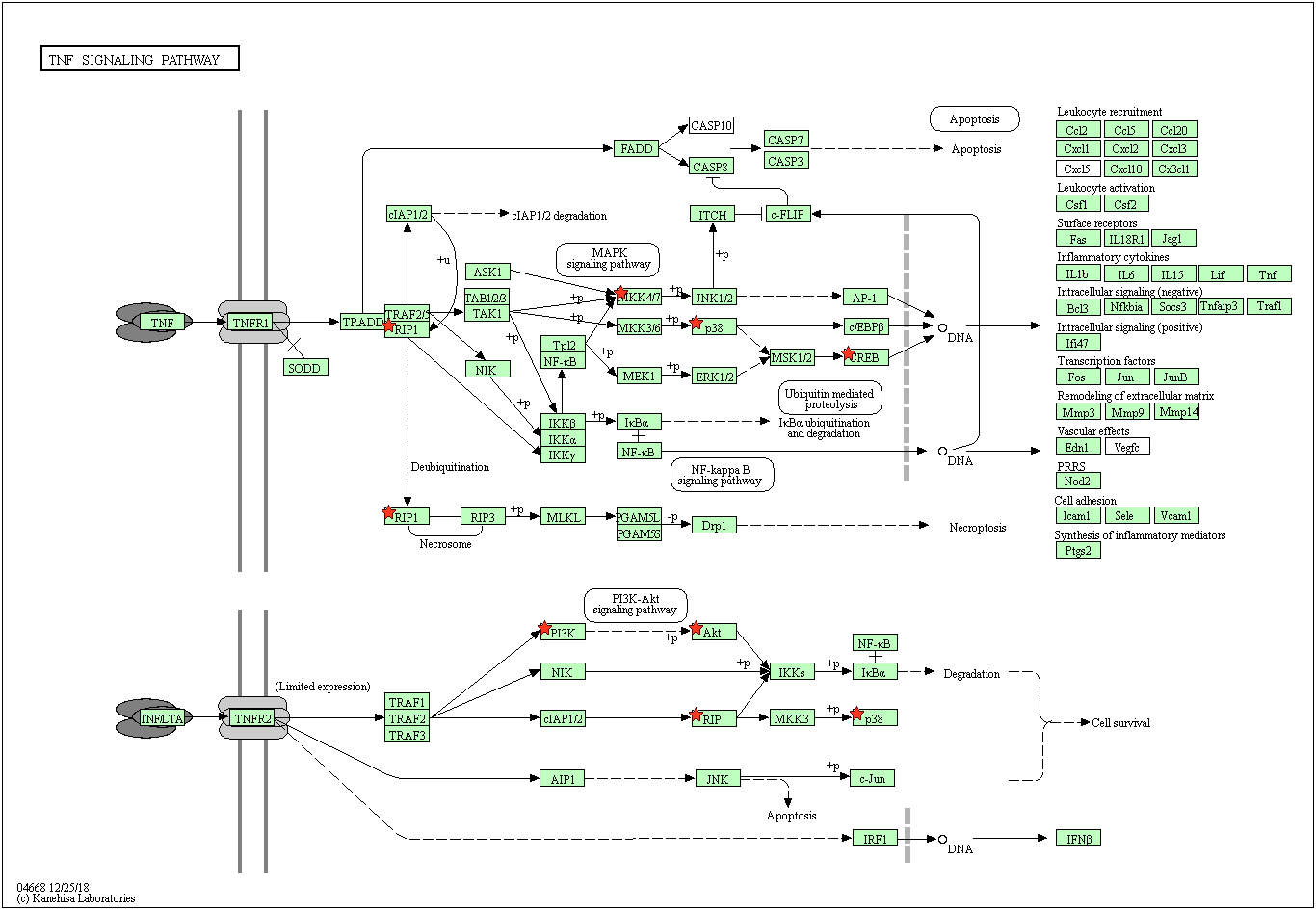

Supplement: S6 Fig — This pathway was enriched in O. maya female WB, the red star symbols indicate the proteins encoded by the upregulated unigenes in females. (TIF) [file pone.0216982.s006.tif]

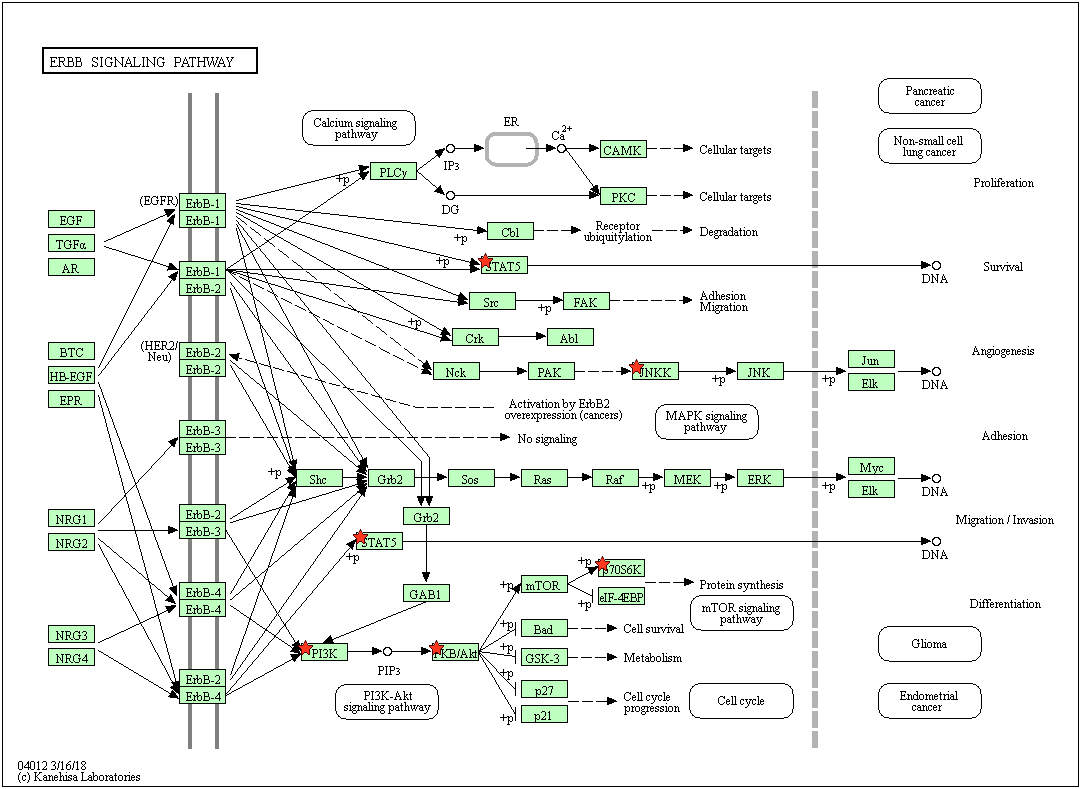

Supplement: S7 Fig — This pathway was enriched in O. maya female WB, the red star symbols indicate the proteins encoded by the upregulated unigenes in females. (TIF) [file pone.0216982.s007.tif]

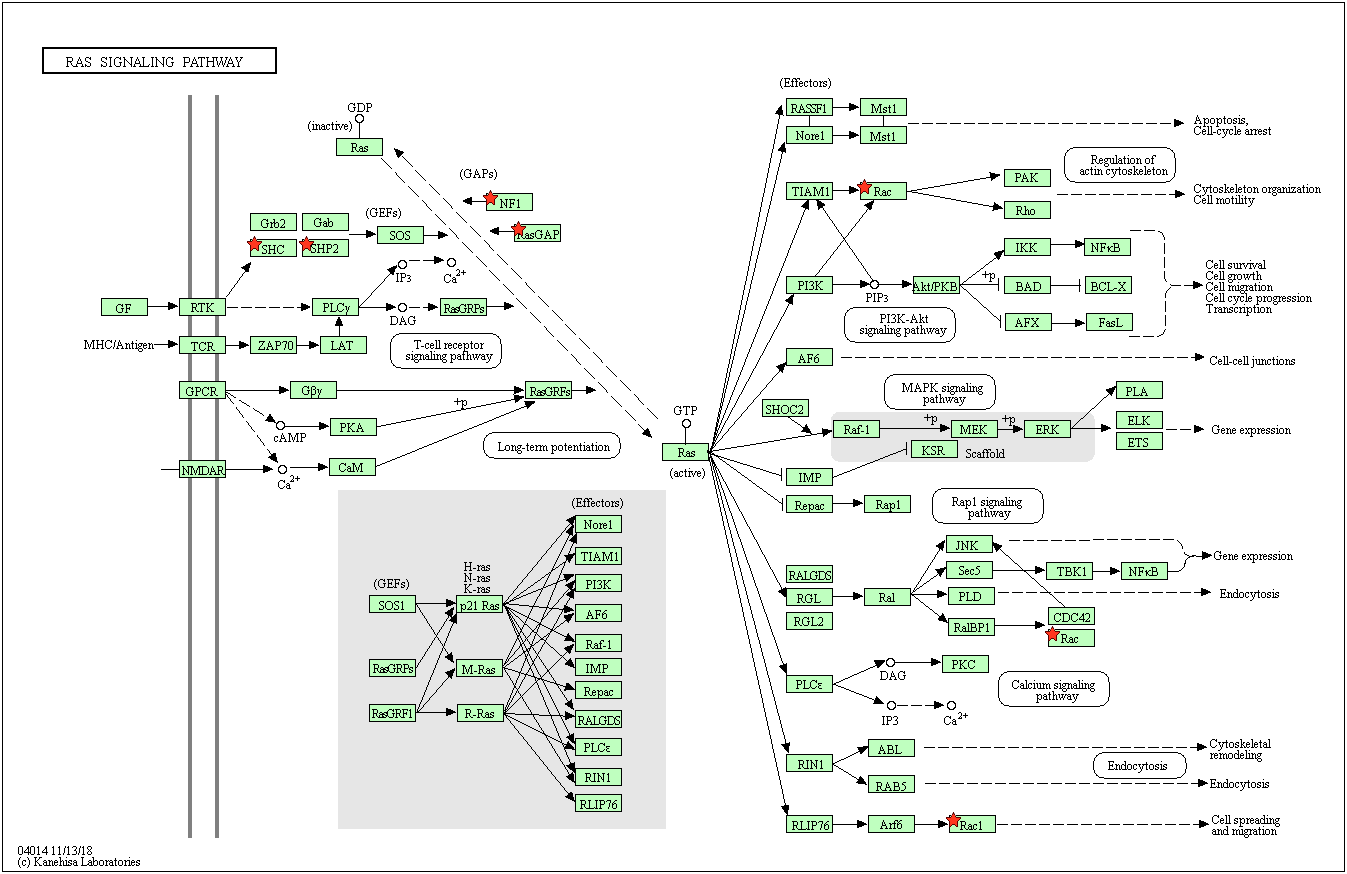

Supplement: S8 Fig — This pathway was enriched in O. maya female WB, the red star symbols indicate the proteins encoded by the upregulated unigenes in females. (TIF) [file pone.0216982.s008.tif]

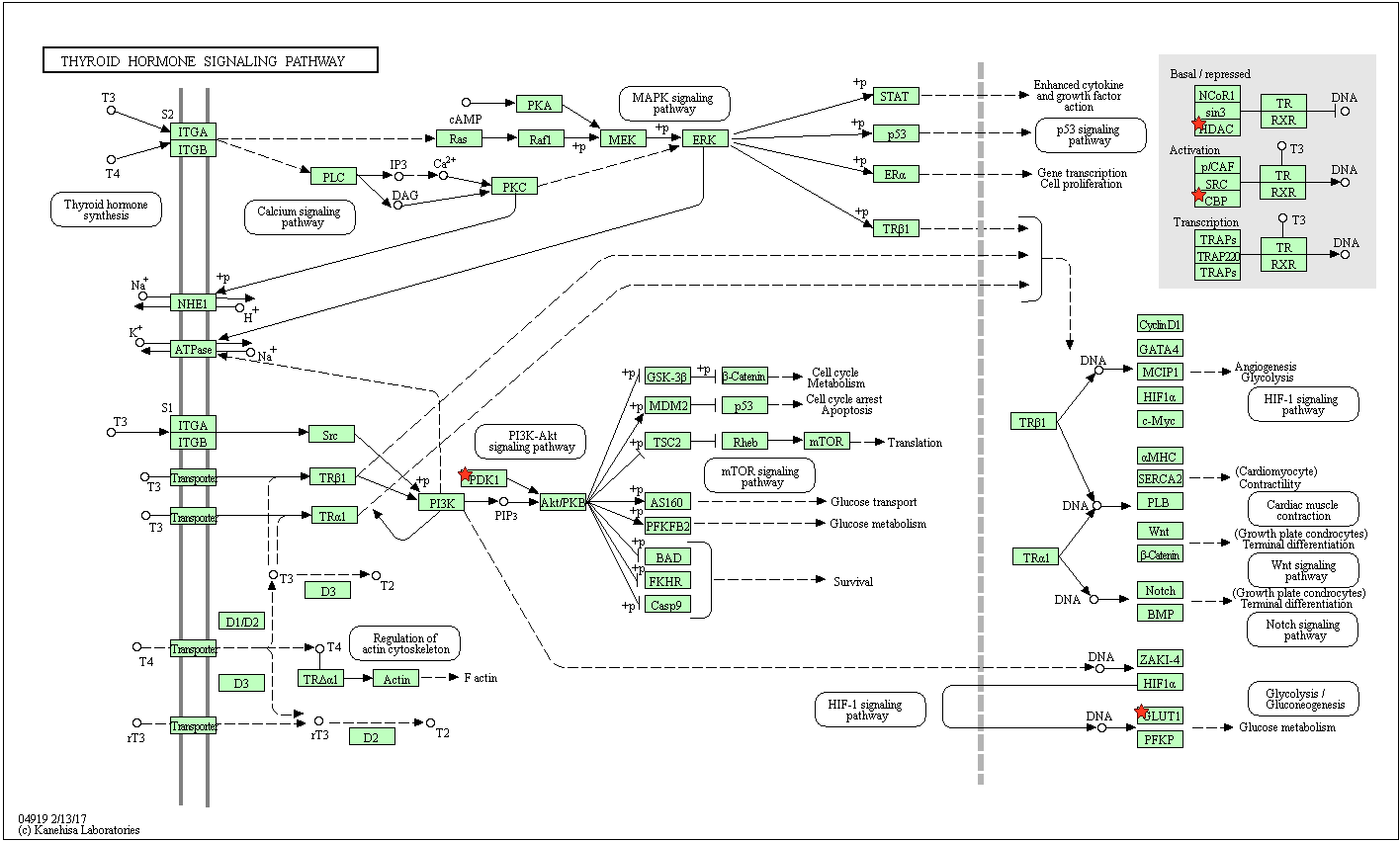

Supplement: S9 Fig — This pathway was enriched in O. maya female WB, the red star symbols indicate the proteins encoded by the upregulated unigenes in females. (TIF) [file pone.0216982.s009.tif]

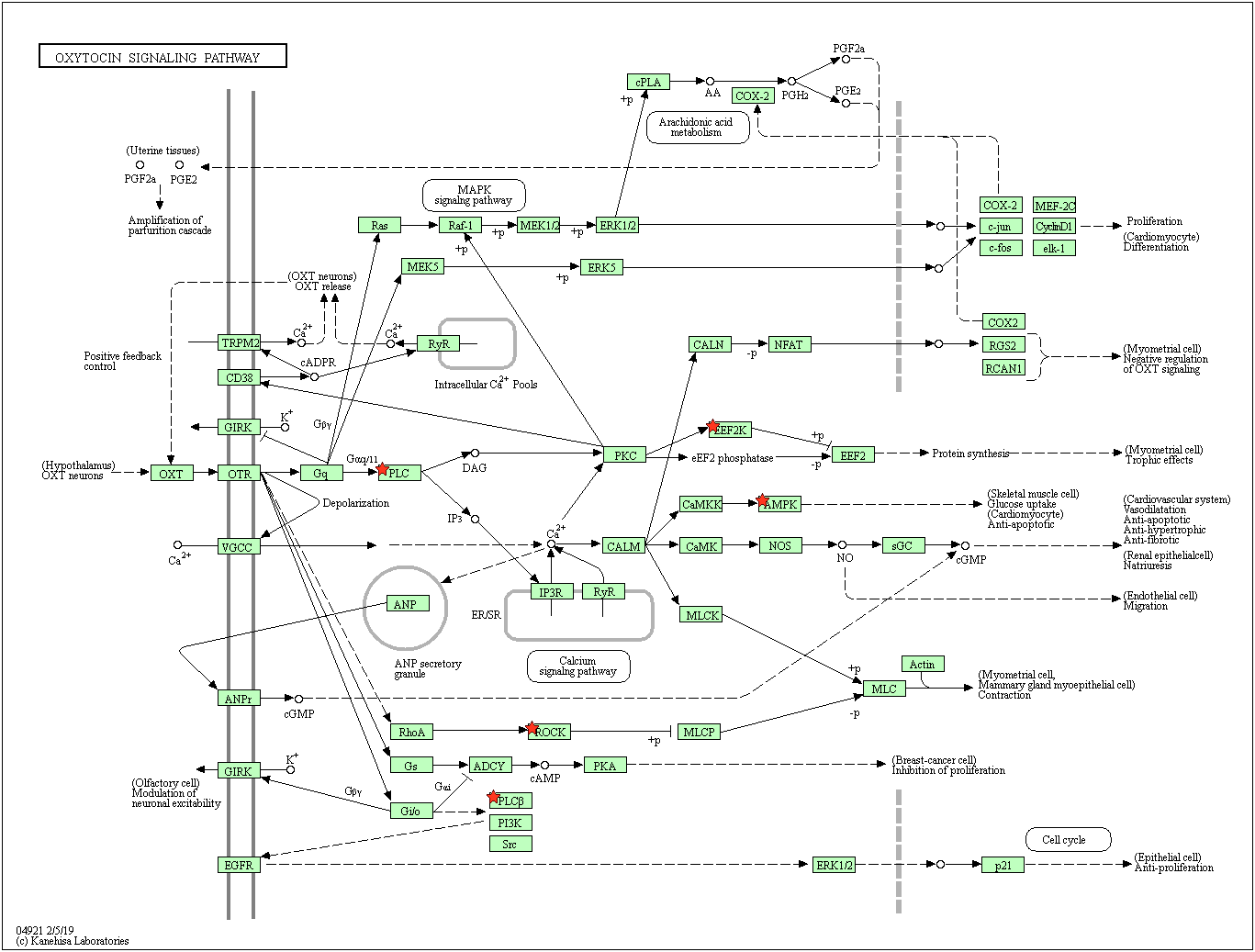

Supplement: S10 Fig — This pathway was enriched in O. maya female WB, the red star symbols indicate the proteins encoded by the upregulated unigenes in females. (TIF) [file pone.0216982.s010.tif]

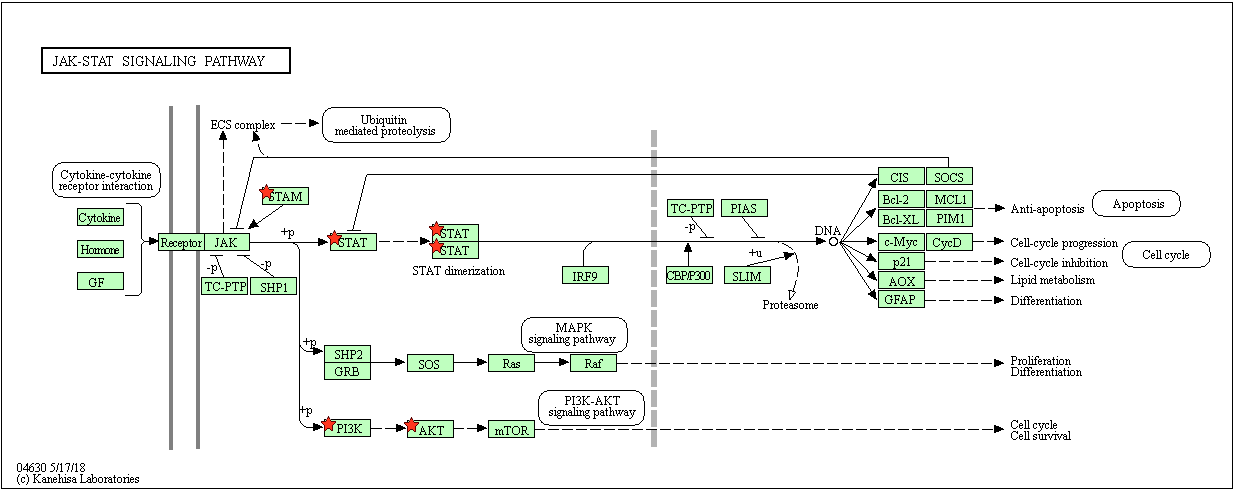

Supplement: S11 Fig — This pathway was enriched in O. maya female WB, the red star symbols indicate the proteins encoded by the upregulated unigenes in females. (TIF) [file pone.0216982.s011.tif]

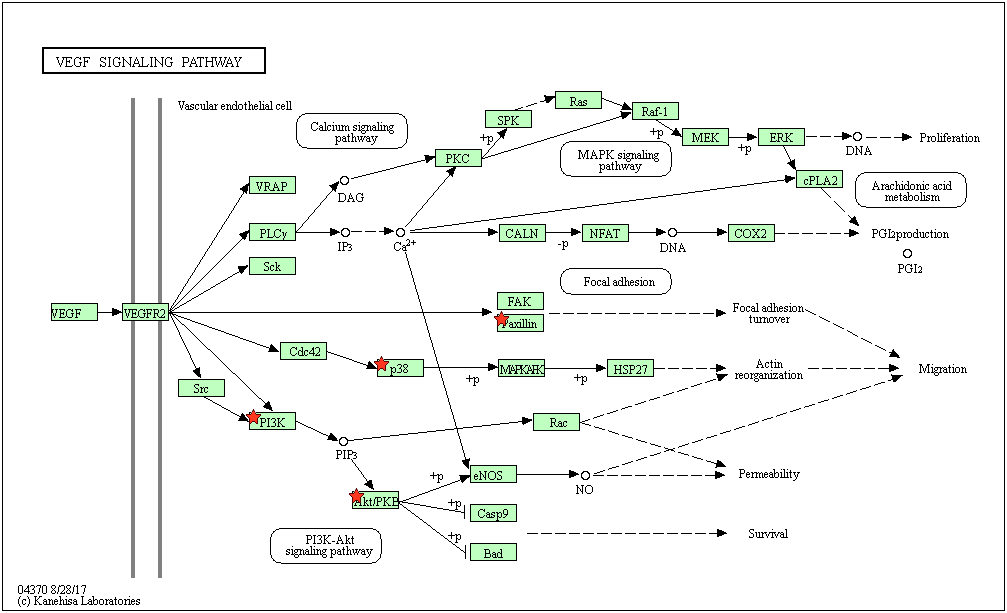

Supplement: S12 Fig — This pathway was enriched in O. maya female WB, the red star symbols indicate the proteins encoded by the upregulated unigenes in females. (TIF) [file pone.0216982.s012.tif]

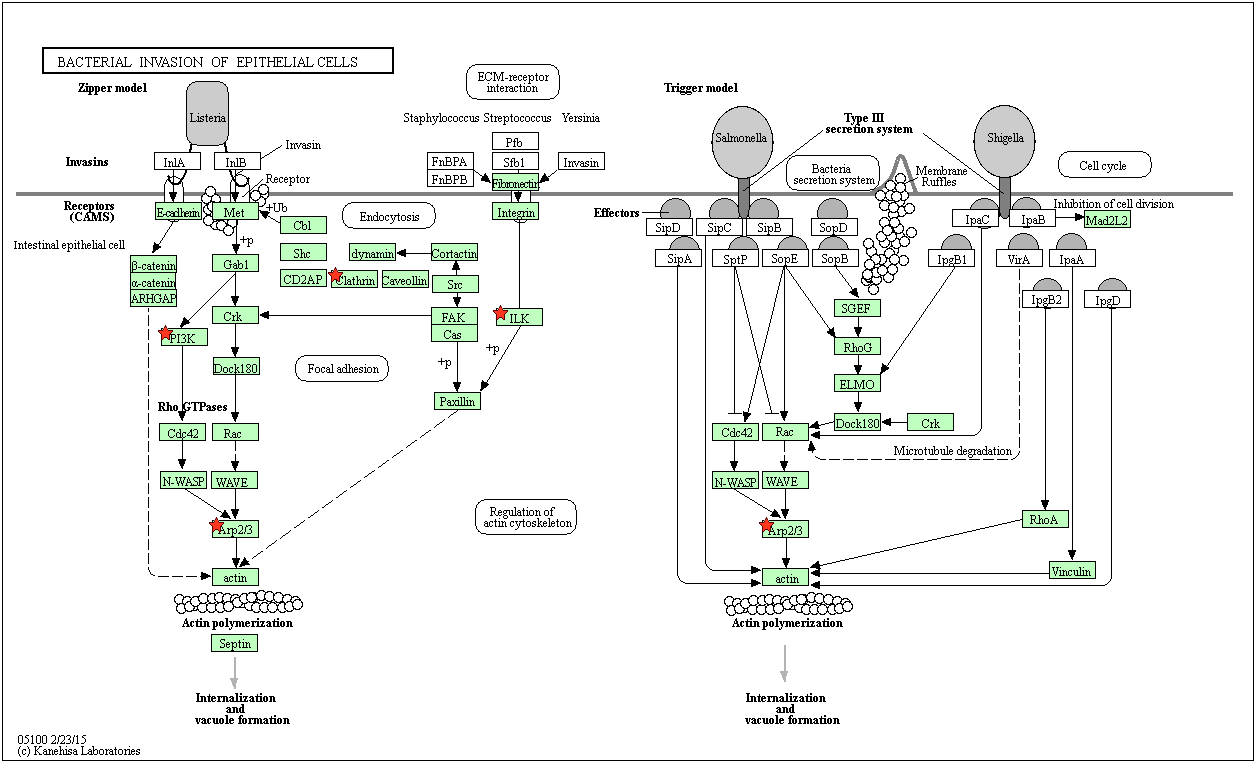

Supplement: S13 Fig — This pathway was enriched in O. maya female WB, the red star symbols indicate the proteins encoded by the upregulated unigenes in females. (TIF) [file pone.0216982.s013.tif]

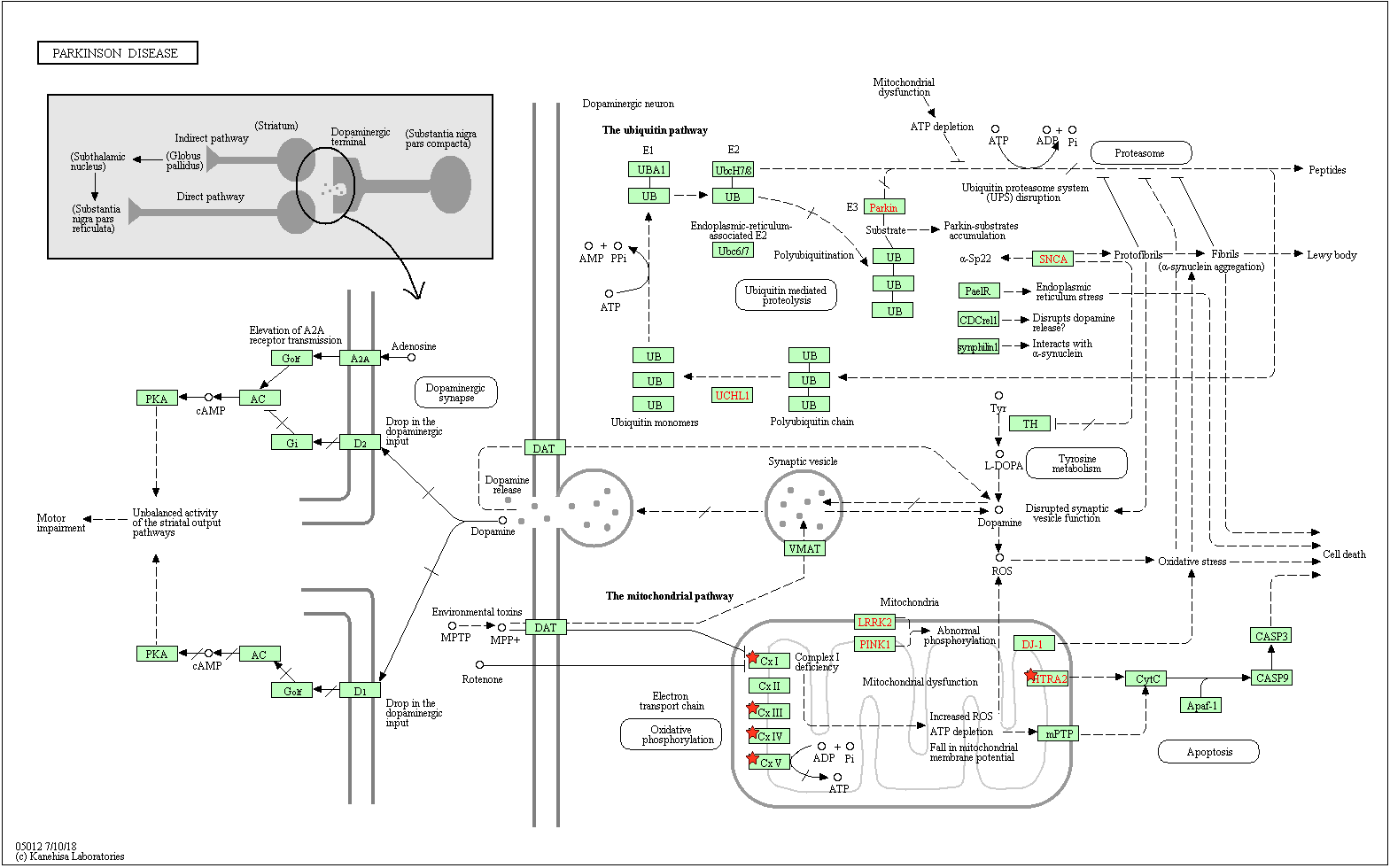

Supplement: S14 Fig — This pathway was enriched in O. maya male WB, the red star symbols indicate the proteins encoded by the upregulated unigenes in males. (TIF) [file pone.0216982.s014.tif]

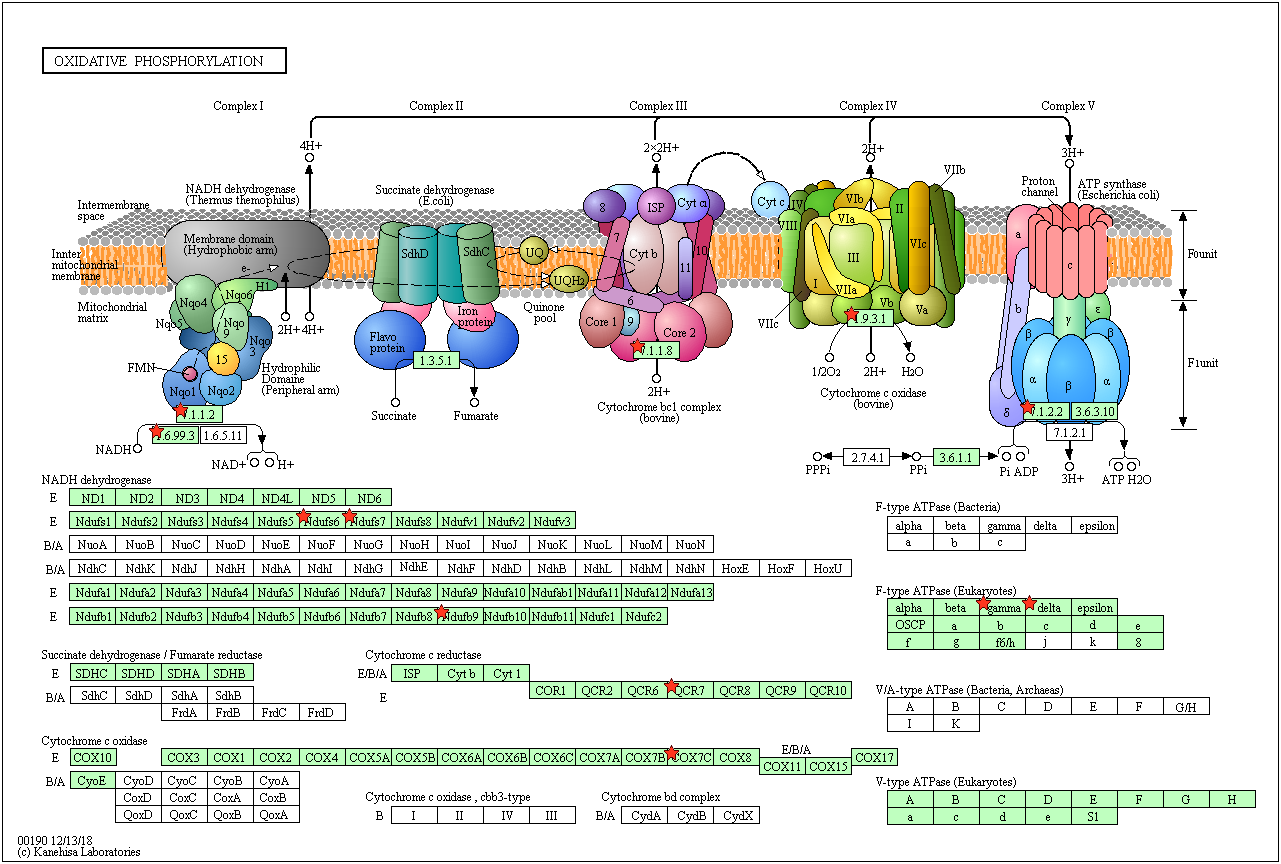

Supplement: S15 Fig — This pathway was enriched in O. maya male WB, the red star symbols indicate the proteins encoded by the upregulated unigenes in males. (TIF) [file pone.0216982.s015.tif]

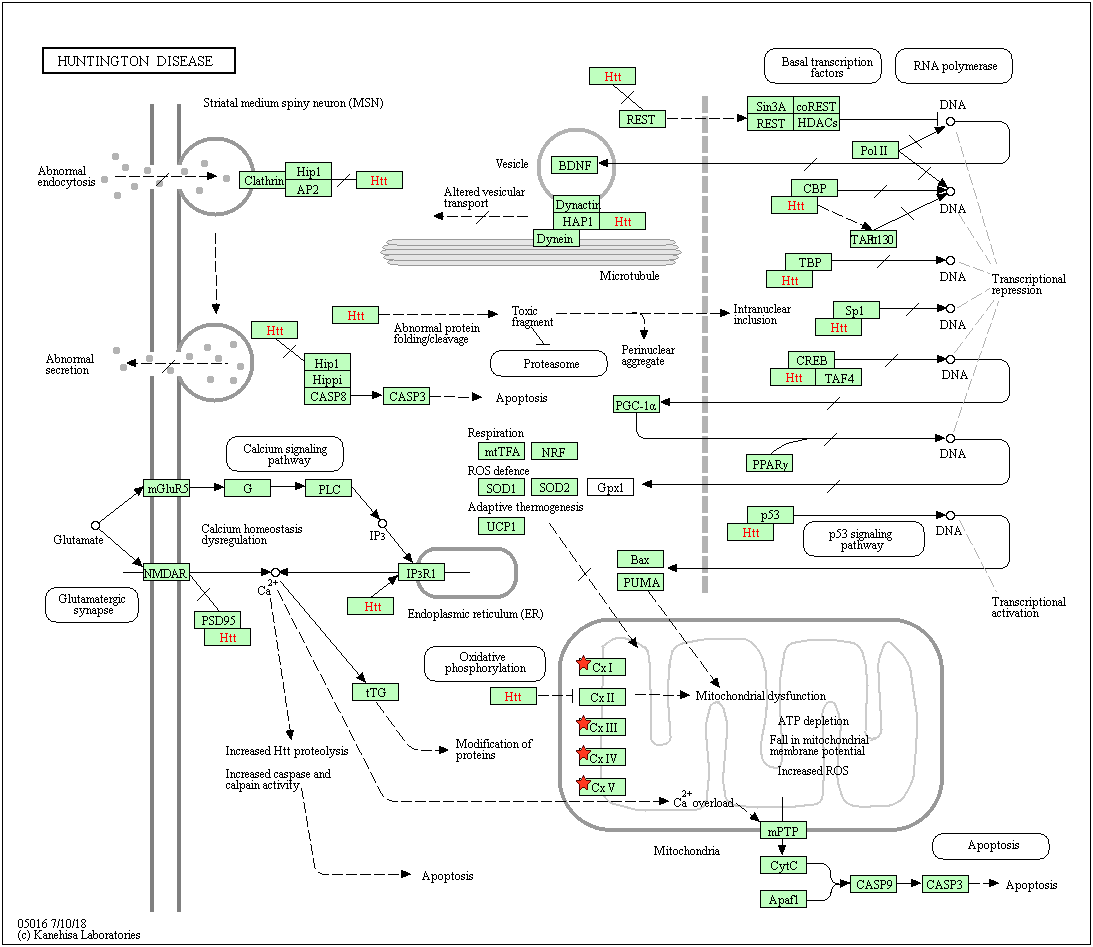

Supplement: S16 Fig — This pathway was enriched in O. maya male WB, the red star symbols indicate the proteins encoded by the upregulated unigenes in males. (TIF) [file pone.0216982.s016.tif]

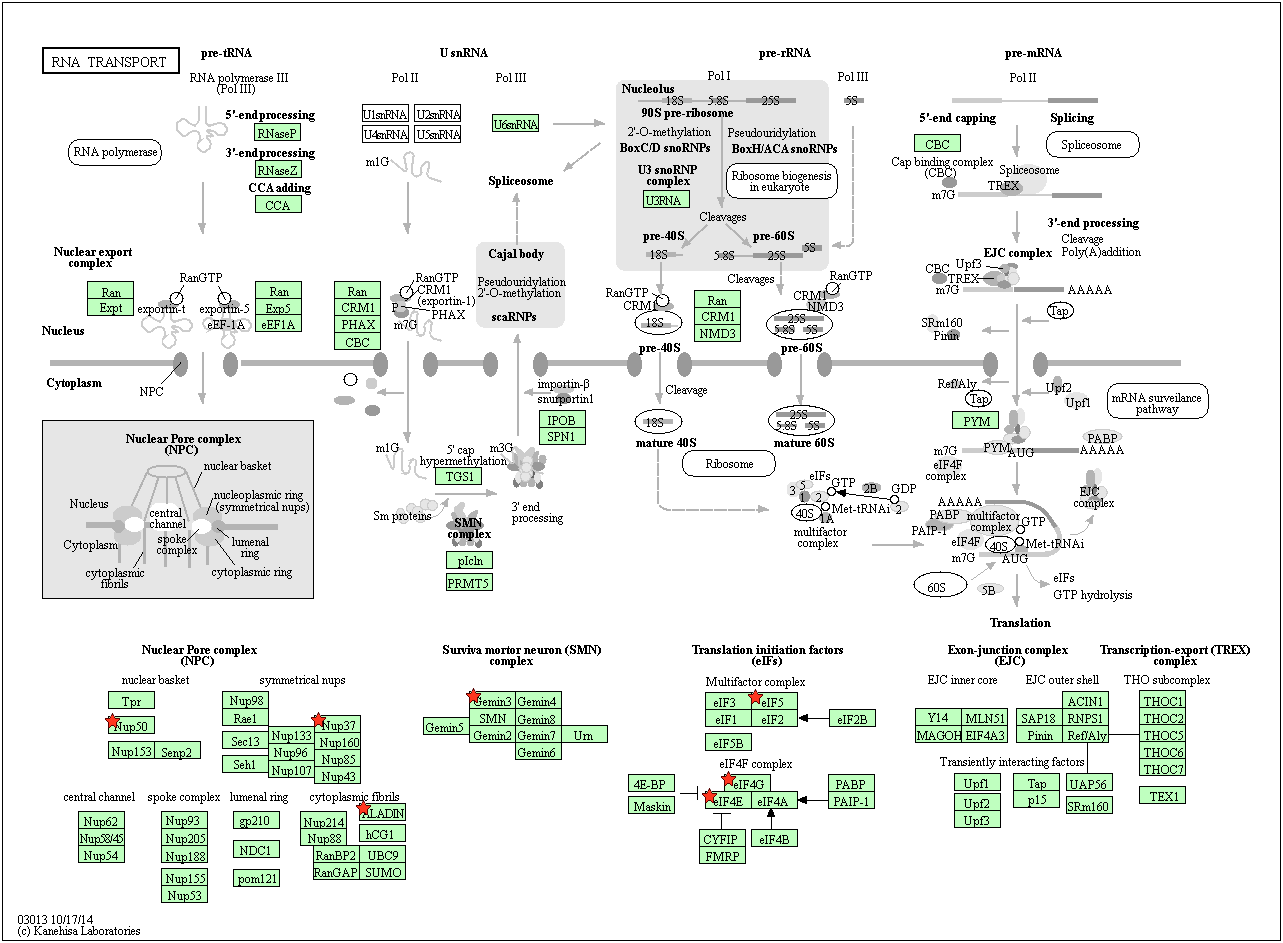

Supplement: S17 Fig — This pathway was enriched in O. maya male WB, the red star symbols indicate the proteins encoded by the upregulated unigenes in males. (TIF) [file pone.0216982.s017.tif]
